# Identification, Expression and IAA-Amide Synthetase Activity Analysis of Gretchen Hagen 3 in Papaya Fruit (*Carica papaya* L.) during Postharvest Process

**DOI:** 10.3389/fpls.2016.01555

**Published:** 2016-10-20

**Authors:** Kaidong Liu, Jinxiang Wang, Haili Li, Jundi Zhong, Shaoxian Feng, Yaoliang Pan, Changchun Yuan

**Affiliations:** ^1^Life Science and Technology School, Lingnan Normal UniversityZhanjiang, China; ^2^The State Key Laboratory for Conservation and Utilization of Subtropical Agro-Bioresources, South China Agriculture UniversityGuangzhou, China; ^3^College of Agriculture and Root Biology Center, South China Agricultural UniversityGuangzhou, China

**Keywords:** auxin, GH3 gene family, papaya, postharvest, ripening, softening

## Abstract

Auxin plays essential roles in plant development. Gretchen Hagen 3 (*GH3*) genes belong to a major auxin response gene family and GH3 proteins conjugate a range of acylsubstrates to alter the levels of hormones. Currently, the role of *GH3* genes in postharvest physiological regulation of ripening and softening processes in papaya fruit is unclear. In this study, we identified seven *CpGH3* genes in a papaya genome database. The CpGH3.1a, CpGH3.1b, CpGH3.5, CpGH3.6, and CpGH3.9 proteins were identified as indole-3-acetic acid (IAA)-specific amido synthetases. We analyzed the changes in IAA-amido synthetase activity using aspartate as a substrate for conjugation and found a large increase (over 5-fold) during the postharvest stages. Ascorbic acid (AsA) application can extend the shelf life of papaya fruit. Our data showed that AsA treatment regulates postharvest fruit maturation processes by promoting endogenous IAA levels. Our findings demonstrate the important role of *GH3* genes in the regulation of auxin-associated postharvest physiology in papaya.

## Introduction

The ripening of fruit is a genetically controlled process that involves complex multi-hormonal crosstalk. Auxin (indole-3-acetic acid, IAA) is a ubiquitous signaling molecule that has a vital role in plant development and growth including cell elongation and division, organ differentiation, embryogenesis, lateral root elongation, shoot architecture, and fruit development (Quint and Gray, 2006; Teale et al., 2008). Along with the hormone ethylene, auxin plays vital roles in many aspects of fleshy fruit development including fruit set and fruit ripening (Jones et al., 2002; Ruan et al., 2012). In tomato, auxin functions to retard fruit ripening through interactions with other hormones, such as ethylene, abscisic acid (ABA), and jasmonic acid (JA) (Su et al., 2015). High concentrations of IAA are required for the biosynthesis of ethylene, which plays a significant role in fruit softening in “melting flesh” peaches at the late ripening stage (Pan et al., 2015).

Auxin coordinates plant development by regulating the expression of auxin response gene families such as *Aux/IAA* (auxin/indole acetic acid), *GH3* (Gretchen Hagen 3), *SAUR* (small auxin up RNA), and *ARF* (auxin response factor) (Abel and Theologis, 1996). A recent transcriptome survey of strawberry fruit revealed dynamic changes in expression of auxin early response gene families during postharvest ripening (Chen et al., 2016). Such studies on the biological functions of auxin response genes help to elucidate the mechanisms underlying the regulation of auxin-mediated fruit ripening.

The protein structures and biological functions of GH3 family members in model plant species have been studied in detail. The *GH3* family genes encode IAA-amido synthetases that are involved in endogenous auxin homeostasis through catalysis of auxin conjugation and by binding free IAA to amino acids (Staswick et al., 2005; Feng et al., 2015). The first GH3 family member was isolated from soybean (*Glycine max*) and subsequently fully identified in the model plant *Arabidopsis thaliana* (Hagen et al., 1984; Takase et al., 2003, 2004). In plants, amino acid conjugation of diverse hormones, including JA, IAA, and salicylic acid (SA), control the concentrations of their bioactive forms to regulate developmental processes (Westfall et al., 2010, 2012). Recently, the crystal structures of the GH3 family members benzoate-specific AtGH3.12/PBS3 and JA-specific AtGH3.11/JAR1 were reported. This analysis found a highly adaptable three-dimensional scaffold for the conjugation of amino acids to diverse acyl acid substrates; it also identified residues forming acyl acid binding sites in the GH3 proteins and residues critical for amino acid recognition (Westfall et al., 2012). The auxin-conjugating enzyme GH3.1 from grapevine (*V. vinifera*) has a similar structure to the GH3 enzymes from *A. thaliana* (Peat et al., 2012). Based on structural details and acyl acid site comparisons, GH3 proteins from different species can be assigned to eight subfamilies. GH3 proteins belonging to subfamilies 1, 2, and 4 show a preference for JA, IAA, and benzoate substrates, respectively. GH3 proteins belonging to subfamilies 3 and 5-8 have no well-defined substrates (Westfall et al., 2010, 2012).

In *A. thaliana*, the gene *WES1* encodes an auxin-conjugating enzyme that plays a role in hypocotyl growth by mediating phytochrome B-perceived light signals (Park et al., 2007). The mutant *ydk1-D*, a T-DNA insertion proximal to *AtGH3.2*, is dominant and displays a dwarf hypocotyl under both light and dark conditions (Takase et al., 2004). Two *GH3* gene homologs in *A. thaliana, DFL1*, and *DFL2*, regulate hypocotyl length and lateral root formation in response to light stimulation (Nakazawa et al., 2001; Takase et al., 2003). Several years ago, homeostasis of miR160 was reported to be involved in the regulation of adventitious root initiation in *A. thaliana* through targeting *AtARF17*, which encodes a negative regulator of *AtGH3.3, AtGH3.5*, and *AtGH3.6* expression (Sorin et al., 2005, 2006; Gutierrez et al., 2009). These results suggest that the *GH3* gene family is involved in adventitious root initiation (Gutierrez et al., 2012). In rice, some *GH3* genes were found to be related to stress responses and developmental regulation. Over-expression of *OsGH3.1* enhances resistance to fungal pathogens by inhibiting cell wall loosening and reducing auxin content (Domingo et al., 2009). In a similar manner to *OsGH3.1*, activation of *OsGH3.13* decreases endogenous auxin content and enhances rice drought tolerance (Zhang et al., 2009). *OsGH3.2* influences drought and freezing tolerance through modulating ABA levels (Du et al., 2012). *OsGH3.5*, a downstream target gene of *OsARF19*, controls rice leaf angles by interacting with the brassinosteroid signaling pathway (Zhang et al., 2015).

In addition to model plant species, the *GH3* gene family has also been identified in fruit plants, including 11 members in citrus (*Citrus sinensis* L.), 15 in apple (*Malus* × *domestica*), 9 in grapevine (*Vitis vinifera* L.), and 2 in longan (*Dimocarpus longan* L.) (Böttcher et al., 2011; Kuang et al., 2011; Yuan et al., 2013; Xie et al., 2015). There is evidence that these *GH3* genes have a role in fruit setting, growth, and ripening (de Jong et al., 2009). In grape, expression of *VvGH3.1* increases at the onset of ripening. Activated IAA-amido synthetase conjugates IAA to amino acids and contributes to the establishment and maintenance of a low IAA concentration, which may accelerate the initiation of ripening (Böttcher et al., 2010). Expression of another grapevine *GH3* gene, *VvGH3.2*, can be induced by treatment with an auxinic compound in pre-ripening berries; this treatment increases the concentration of IAA-Asp and decreases the concentration of free IAA (Böttcher et al., 2011). These results indicate that GH3 proteins have various roles in controlling fruit ripening in both auxin-dependent and JA-dependent manners.

As a climacteric fruit, papaya exhibits rapid softening and has a short-term shelf life, which significantly limits its market value (Jain et al., 2011). The elucidation of how endogenous hormones function in postharvest decay under different conditions is therefore of importance not only to plant biologists but also to agronomists (Gomez-Lobato et al., 2012; Chen et al., 2016). However, little is known of the roles of endogenous auxin in the postharvest maturation of papaya fruit. Our study provides comprehensive information on *GH3* gene expression patterns in different tissues and on the enzyme activities of IAA-amido synthetases under different postharvest conditions. These data will be important to the development of new postharvest strategies for papaya.

## Materials and methods

### Plant materials and treatments

We used the *Carica papaya* cultivar “Sunrise” in this study. Two-years-old trees planted in a 3 m × 3 m arrangement were provided with standard drip irrigation and fertilizer applications. The experimental field at Lingnan Normal University field experimental station in Zhanjiang City (Guangdong Province, China) has a tropical climate and experiences oceanic monsoons; it has an average daily temperature of 22.8°C, with a minimum of 15.7°C and a maximum of 28.8°C. The total yearly rainfall ranges between 1100 and 1800 mm. Five tissue samples were used for tissue-specific expression pattern analysis. In detail, the shoot, leaf, root samples were selected from 1-year-old papaya trees. The fruit samples were harvested from fruits at the color break stage (5% ≤ peel color ≤ 15% yellow) of 2-years-old trees. The flower samples were selected from mature flower with opened petals of 2-years-old trees. The selected were washed with deionized water, and then dipped in 75% (w/w) alcohol for 45 s to eliminate potential microbes.

### Measurement of fruit firmness, weight loss, total soluble solids, and titratable acidity

A hand-held fruit firmness tester (GY-J, Top Instrument Co, Ltd, Zhejiang, China) with an 8-mm probe attached to a digital force gauge was used to determine papaya firmness. The mean of five independent measurements was calculated for each papaya fruit and expressed in Newtons (N). Weight loss was estimated by measuring the weight of the whole papaya fruit from the beginning to the end of different storage periods. Weight loss was expressed as the percentage of initial weight. Fruits were packed in commercial boxes and stored at 20 ± 1°C and 85–90% relative humidity. The time points after harvest of 0, 5, 10, 15, 20, and 25 days were defined as postharvest stages 1–6, respectively.

Fruit pulp (5.0 g) from three replicates of five independent fruit for each treatment was prepared using a mortar and pestle in 50.0 mL distilled water. The homogenate was centrifuged at 15,000 × g for 20 min at 4°C, and then the supernatant was used to measure total soluble solid content (%) with a hand-held refractometer (J1-3A, Guangdong Scientific Instruments, Guangzhou, China).

### Respiration rate determination and pericarp color characteristics assay

The respiration rate was determined by conducting an infrared analysis. Five replicate groups of three papaya fruits from each treatment were weighed, and then sealed in a 2.4-L container at 25°C. An infrared gas analyzer (Li-6262 CO_2_/H_2_O analyzer, LI-COR, America) was used to monitor CO_2_ concentration in the container. A colorimeter (Minolta, model CR-400, Tokyo, Japan) was used to determine the color characteristics of the papaya pericarp. Three independent points in the equatorial region of the papaya fruit skin were chosen to determine color characteristics. The method for assessing color characteristics has been described previously (Liu et al., 2014).

### Identification of *GH3* genes in *Carica papaya*

*A. thaliana* GH3 protein sequences were used in a BLAST search of the *C. papaya* in Phytozome 10.1 database (http://phytozome.jgi.doe.gov). The sequences of the AtGH3 proteins are shown in Table S1. The maximum *e*-value acceptable in the BLAST search for identifying GH3 members was “10^−3^.” The Hidden Markov Model (HMM) profiles of the *GH3* gene family (Pfam: 03321, *GH3* auxin-responsive promoter) were used to identify the candidate sequences (http://pfam.xfam.org/). All the obtained sequences were sorted as unique sequences for a further protein domain search using InterProScan Sequence Search (http://www.ebi.ac.uk/Tools/pfa/iprscan/). Motifs characteristic of CpGH3 proteins were investigated by Multiple Expectation Maximization for Motif Elicitation (MEME 4.10.2) web server (http://meme.nbcr.net/meme/cgi-bin/meme.cgi).

### Phylogenetic tree construction and intron-exon structure analysis

ClustalW (http://www.ebi.ac.uk/Tools/msa/clustalw2/) was used to perform multiple sequence alignments on the identified CpGH3 protein sequences with the default parameters. Subsequently, GeneDoc (http://www.nrbsc.org/gfx/genedoc/) was used to visualize the alignments. A phylogenetic tree was built using the MEGA5.1 software (http://www.megasoftware.net/mega5/mega.html) by neighbor-joining method for 19 *A. thaliana* and seven *C. papaya* GH3 protein sequences. Bootstrap values were calculated from 1000 iterations. Exon-intron organization of *CpGH3* genes were determined by comparing the coding sequences with their corresponding genomic sequences downloaded from the database Phytozome 10.1.

### *Cis*-element analysis

The 1500-bp promoter regions of *CpGH3* genes were obtained and downloaded from Phytozome 10.1. An auxin response element (AuxRE: TGTCTC), an ABA responsive element (ABRE: YACGTGK), a SA responsive element (SARE: TGACG), a JA responsive element (JERE: AGACCGCC), and an ethylene responsive element (GCC: AGCCGCC) were used to scan the promoter regions of the *CpGH3s*. The results were confirmed using PLACE software (http://www.dna.affrc.go.jp/PLACE/).

### RNA isolation and quantitative RT-PCR

Total RNAs from roots, shoots, leaves, flowers, and fruits from the different treatment groups were isolated with a plant RNeasy Mini kit (Qiagen, Hilden, Germany) according to the manufacturer's instructions. Genomic DNA contamination was removed using DNase I (TaKaRa, Dalian, China). The *CpActin* gene (evm.model.supercontig_18.238) was used as an internal standard to calculate relative fold differences based on comparative cycle threshold (2^−Δ*ΔCt*^) values. The qRT-PCR was performed as described previously (Yang et al., 2015). The primer sequences for the qRT-PCR were designed using Primer Premier 5 software and are listed in Table S2. The limit of detection and the amplification efficiency of the qRT-PCR was performed using 10-fold serial dilution of cDNA isolated from one sample (leaves), which was used to create the standard curve. The slopes and correlation coefficients of the standard curves were used to calculate the PCR efficiency primer pairs. In our experiment, the value of PCR efficiency (E) for each primer pair was calculated by formula: E = POWER (10, 1/slope)-1. The value of PCR efficiency for each primer pair was between 0.9 and 1.1. The standard curves for absolute quantification RT-PCR of CpGH3 genes were showed in Figure S1. The fruits used in tissue-specific expression experiment are right before harvest, 150 days post-fertilization. To avoid the effects of environmental stress, the fruit samples were collected from a large number of independent papaya trees that were distributed throughout the test field. Five fruits from these samples were randomly assigned to each group for the qRT-PCR test.

### Tissue homogenization and enzyme activity assay

One gram of pericarp and sarcocarp was excised from each papaya fruit and homogenized in 1 mL extraction buffer (50 mM Tris-HCl buffer containing 2 mM EDTA, protease inhibitor, and 5 mM 2-mercaptoethanol, pH 7.6) using a mortar and pestle. The homogenate was centrifuged at 12,000 × g for 30 min, and the supernatant was used for the enzyme activity assay. The assay was performed following the procedure described by Ostrowski and Jakubowska (2013). Briefly, enzyme activity was determined in a total volume of 15 μL buffer containing 50 mM Tris-HCl, pH 8.6, 2 mM IAA, 50 mCi mmol^−1^, 10 mM aspartic acid, 5 mM ATP, 5 mM MGCl_2_, and 2 mM DTT. Then, 6 μL of the supernatant fluid was used to start the reaction.

### Protein purification and enzyme assay

The coding regions of *CpGH3.1a, CpGH3.1b, CpGH3.5, CpGH3.6*, and *CpGH3.9* were amplified by PCR from a papaya cDNA template using gene-specific primers with additional restriction sites. The primer sequences are listed in Table S4. These PCR products were closed into restriction sites of a pET-21(a)-His vector to generate GH3-His fusion protein. These five construct were expressed in *Escherichia coli* (DH5α) according to the manufacturer's protocol (Takara, Dalian, China). The expressed fusion proteins were purified with His GraviTrap columns (GE Healthcare, Little Chalfont, UK) and the protein concentration of each sample was determined using a Protein Quantification Kit-Rapid (SIGMA-ALDRICH, Shanghai, China). The assays about enzyme activity with IAA substrates were performed according to Ostrowski's description (Ostrowski and Jakubowska, 2013).

### AsA application, IAA content and ethylene production rate measurements

For AsA application, fruits were randomly distributed two groups. One group was assigned to water (control treatment) and 250 mM AsA for 10 min. After treatment, the fruit were air-dried, packed into polyethylene bags (0.03 mm), and maintained at room temperature with 75% relative humidity. Three replicates were performed for each treatment.

For IAA content measurement, fruit samples were collected and washed three times in ddH_2_O, and then blotted dry with a paper towel. A sample (50 mg) was obtained from each fruit. For IAA content measurement, 500 pg of ^13^C6-IAA was added to each sample as an internal standard. ProElu C18 (http://www.dikma.com.cn) was used to purify the samples. Five independent biological replicates of each sample were used in our experiment, and IAA contents were determined using a FOCUS GC-DSQII (Thermo Fisher Scientific Inc., Austin, TX, USA). The experiment of IAA measurement was performed according to Liu's protocol (Liu et al., 2012).

For ethylene production measurement, two fruits in each of three replicates were placed in a 1000 mL flask for 1 h. Then, 1 mL space samples were collected and ethylene concentrations measured by flame ionization gas chromatography using a SP 6800 gas chromatograph fitted with a GDX-502 column held at 90°C.

### Statistical analysis

Differences between different groups of samples were calculated with Student's *t*-test at a significance level of 0.05 in software Excel. All the expression analysis was performed for five biological repeats and the values shown in figures represent the average values of five repeats, and the data are expressed as the mean and standard deviation (mean ± SD).

## Results

### Identification and phylogenetic analysis of the *GH3* gene family in *C. papaya*

Seven *GH3* genes were identified in *C. papaya*; these genes were all named according to the phylogenetic relationship between *C. papaya* and the model plant *A. thaliana*. The information on the *CpGH3* genes, including gene names, IDs, intron numbers, ORF lengths, and deduced polypeptide parameters, is summarized in Table S3. The sizes of the deduced CpGH3 proteins varied from 421 amino acids (CpGH3.9) to 607 amino acids (CpGH3.1a and CpGH3.1b), molecular masses varied from 47.62 to 68.69 kDa, and predicted isoelectric points varied from 5.61 (CpGH3.1b) to 6.86 (CpGH3.5).

**Figure 1:**
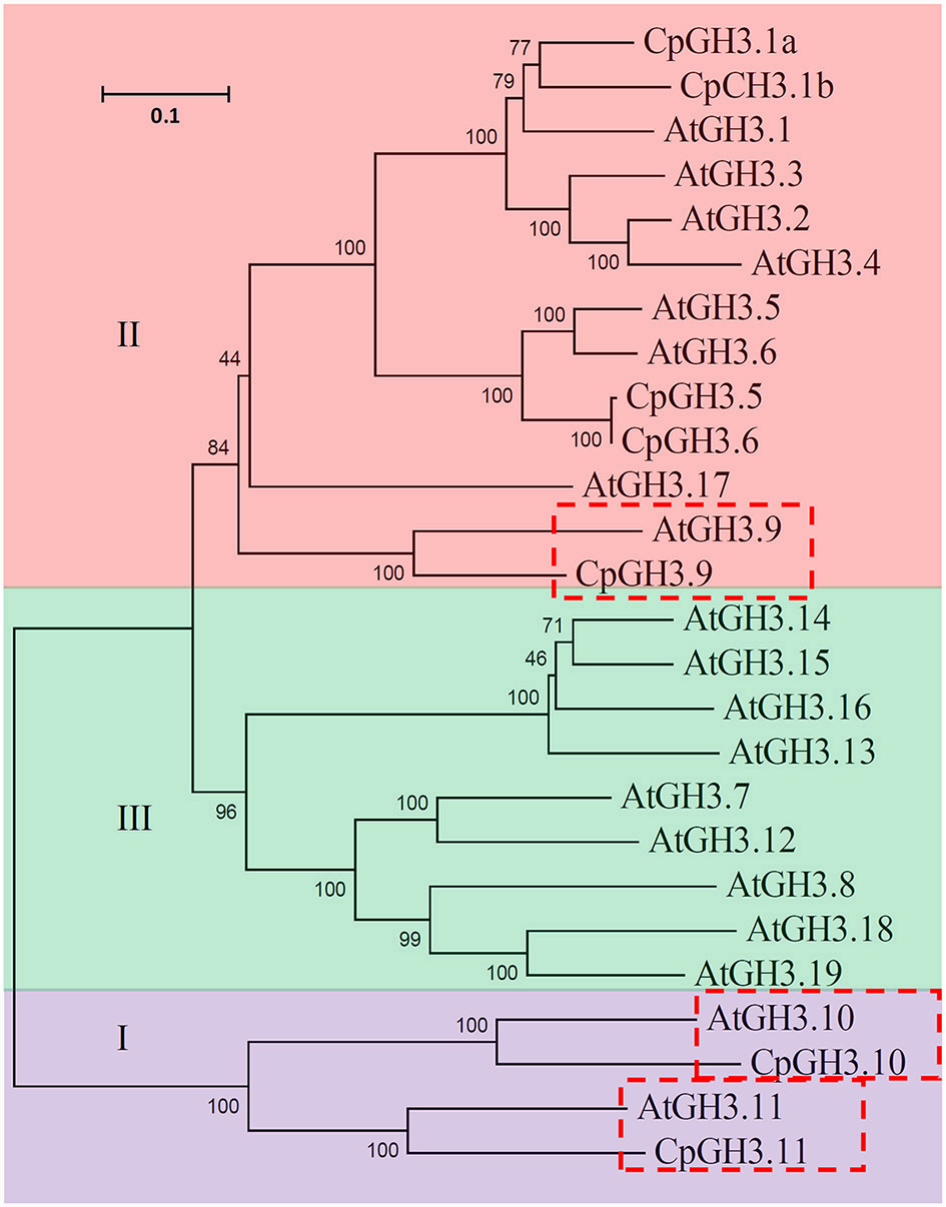
**Phylogenetic relationship analysis of CpGH3 genes**. An unrooted phylogenetic tree was constructed for 19 *Arabidopsis* GH3 and seven papaya GH3 protein sequences using ClustalW using the neighbor-joining method. Homologous pairs are indicated by the red boxes.

To investigate the relationship of the *GH3* genes of *C. papaya* and *A. thaliana*, a phylogenetic tree was constructed for 19 *AtGH3* genes and the seven *CpGH3* genes. The results indicated that the *GH3* gene family could be grouped into three major subfamilies, I, II, and III. Seven *CpGH3* genes were grouped into subfamilies I and III; no *CpGH3* gene belonged to subfamily III. Based on the phylogenetic relationship, three homologous pairs with high bootstrap values (>99) were identified between *CpGH3* and *AtGH3* families: *AtGH3.9*/*CpGH3.9, AtGH3.10*/*CpGH3.10*, and *AtGH3.11*/*CpGH3.11* (Figure 1). The exon-intron structures in the *CpGH3* gene family varied with one to three introns (Figure S2).

### Multiple sequence alignments and acyl acid substrate prediction

Next, we performed a multiple sequence alignment of the 19 AtGH3 protein sequences and seven CpGH3 protein sequences. The alignment results showed that the CpGH3 proteins all contained a highly conserved GH3 domain (Figure S3). Additionally, the MEME tool mapped three conserved motifs to all CpGH3 proteins (Figure S4). The motif similarities with AtGH3 proteins indicated that the functions of the CpGH3 proteins could be predicted by this sequence comparison.

**Figure 2:**
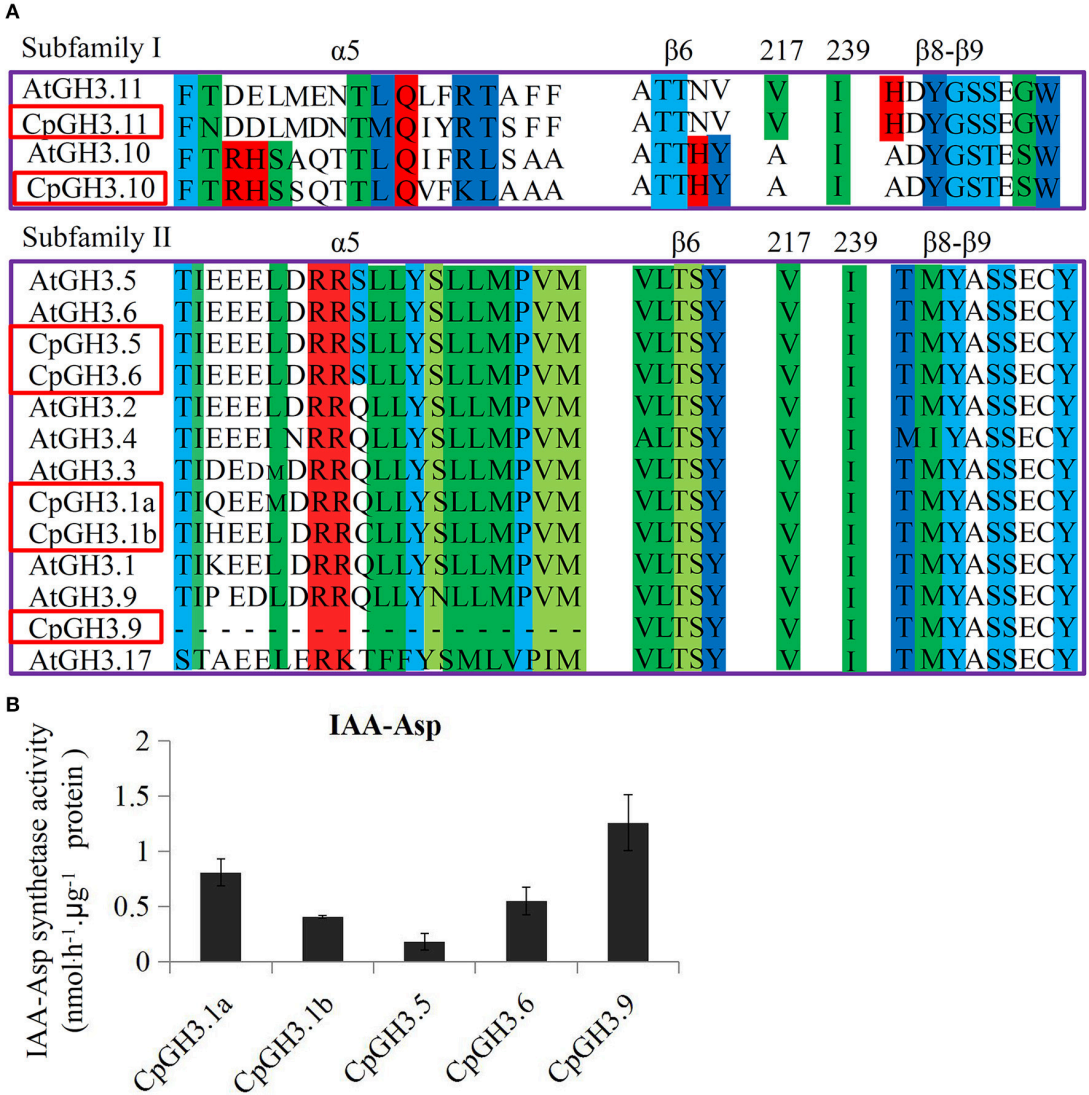
**Phylogenetic relationship analysis of GH3 family and sequence-based grouping of plant GH3 proteins based on acyl acid binding site residues. (A)** CpGH3 and AtGH3 proteins are color coded according to their subgroup. Sequences corresponding to the residues of the α5 and α6 motifs, to residues 217 and 299 of AtGH3.12, and to the β8 and β9 motifs are indicated (Westfall et al., 2012). Conserved residues are highlighted by colored boxes. **(B)** Analysis of IAA-amido synthetases activities of CpGH3 proteins. All five GH3 proteins were purified from *E. coli*, and the *E. coli* with empty vector was used as negative control. The activities have been normalized to μg of protein.

Based on the conserved amino acid residues, most CpGH3 proteins could be assigned to the conjugate protein groups identified in *A. thaliana*. CpGH3.11 was grouped into subfamily I that includes AtGH3.11/JAR1 and which shows enzymatic activity with JA. CpGH3.1a, CpGH3.1b, CpGH3.5, CpGH3.6, and CpGH3.9, like AtGH3.1-6, AtGH3.9, and AtGH3.17, had IAA as the acyl acid substrate (Figure 2A). CpGH3.10 in subfamily I has no currently identified substrates (Westfall et al., 2012). Furthermore, five putative IAA-synthetases, CpGH3.1a, CpGH3.1b, CpGH3.5, CpGH3.6, and CpGH3.9, were chose to analyze activity of the IAA amido synthetases. These GH3 proteins were purified from *E. coli* (Figure S5). Our data showed that these proteins are IAA-amido synthetases (Figure 2B).

### Tissue-specific expression patterns of *CpGH3* genes

**Figure 3:**
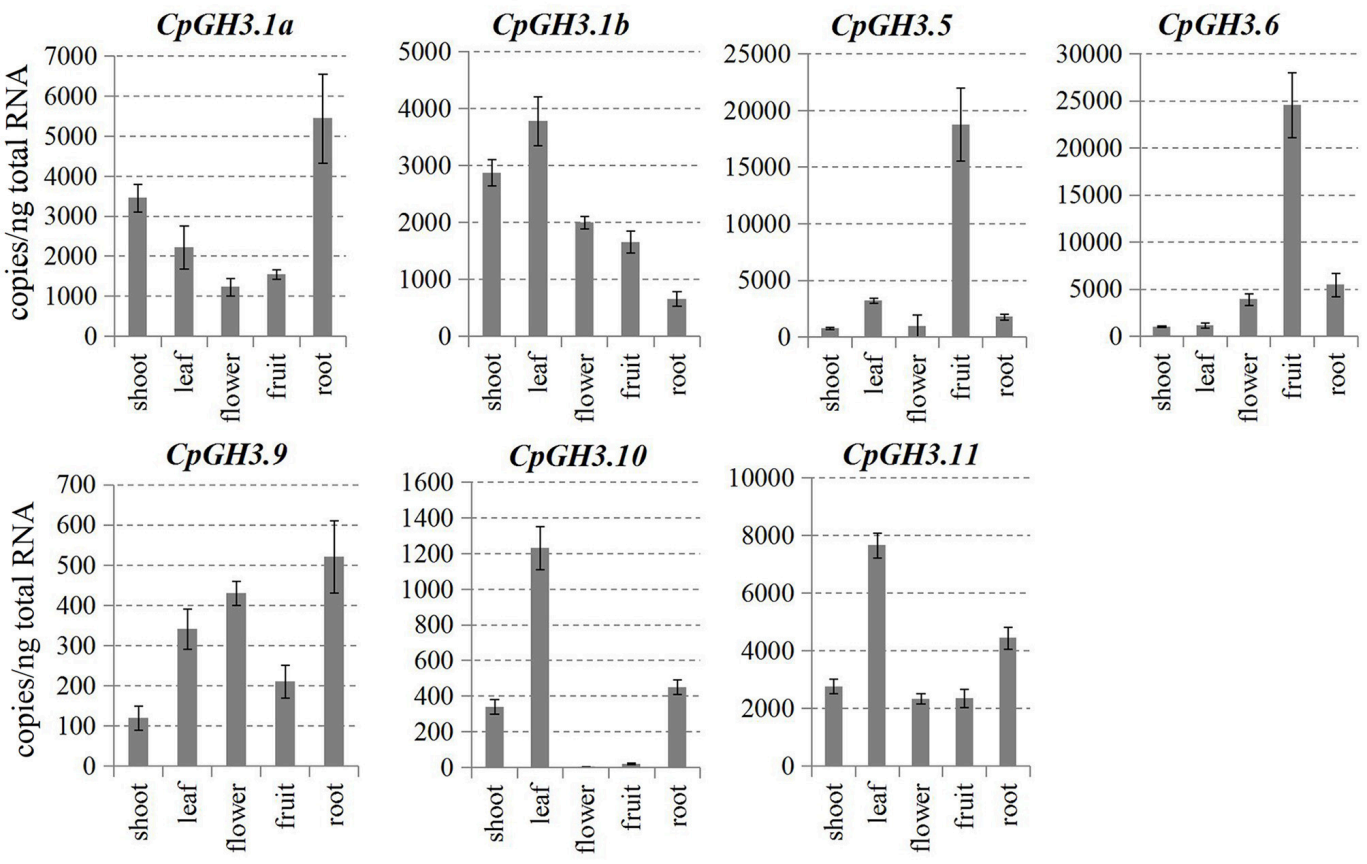
**Tissue-specific expression patterns of *CpGH3* genes**. Expression patterns of *CpGH3* genes were analyzed by absolute quantification RT-PCR; tissue samples were collected from leaf, shoot, root, flower, and fruit of 2-year-old plants. RNA from each tissue was isolated for qRT-PCR. The data were obtained from five independent repeats, and standard deviations are shown with error bars.

Tissue-specific expression of the *CpGH3* genes was analyzed by absolute quantification RT-PCR. Transcripts of all *CpGH3* genes were detectable in different tissues and organs in papaya. *CpGH3.1a* and *CpGH3.9* were more highly expressed in the roots than in other organs; *CpGH3.10* and *CpGH3.11* were highly expressed in the leaves. Interestingly, the transcript level of *CpGH3.10* was very low in fruit, whereas *CpGH3.5* and *CpGH3.6* predominantly expressed in fruit (Figure 3).

### *Cis*-element analysis and hormone responsive expression of *CpGH3* genes

Several phytohormone-related *cis*-elements, such as AuxRE, SARE, and JERE, have been identified in plants (Ulmasov et al., 1997; Paterson et al., 2004; Osakabe et al., 2014). Here, we scanned the 1500-bp upstream promoter regions of *CpGH3* genes for phytohormone-related *cis*-elements. Several such elements were identified, namely ABRE, AuxRE, and SARE; no JERE sequences were found in these promoter regions. One AuxRE and one SARE element were present in the *CpGH3.1a* promoter; three SAREs were present in the *CpGH3.1b* promoter; two AuxREs were present in the *CpGH3.5* promoter; two ABREs were present in the *CpGH3.6* promoter; and two ABREs, two AuxREs, and two SAREs were present in the *CpGH3.11* promoter. The numbers of these hormone-related *cis*-elements in the upstream 1.5-kb regions of *CpGH3* genes are listed in Table S4.

### *CpGH3* expression and IAA-amido synthetase activity at different postharvest stages

**Figure 4:**
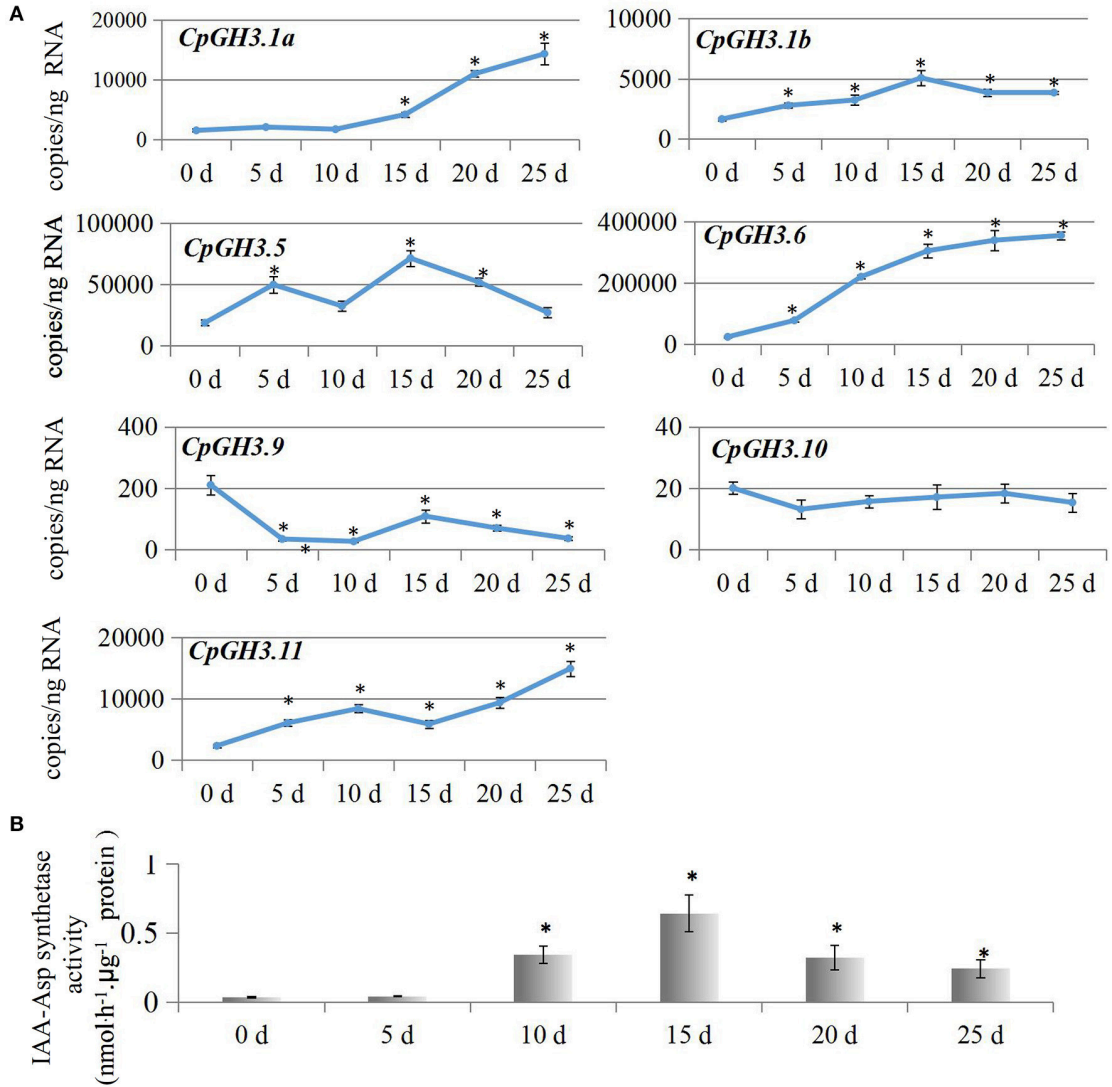
**Analysis of *CpGH3* gene expression patterns and IAA-amido synthetase activities during different postharvest stages**. **(A)** Analysis of expression of *CpGH3* genes during different postharvest stages. **(B)** Enzymatic activities of IAA-amido synthetase. The data were obtained from by five independent repeats, and standard deviations are shown with error bars. Significant differences (*P* < 0.05) between “stage 1” and the other postharvest stages are indicated by an asterisk.

Genetic studies have revealed that fruit ripening and softening is mediated by auxin-responsive genes in an auxin homeostatic process (Pan et al., 2015). To elucidate the functions of *CpGH3* genes during the postharvest period, we analyzed the changes in expression levels at six different postharvest stages. Our analysis indicated that expression of most *CpGH3* genes changed significantly during fruit ripening and softening. *CpGH3.1a, CpGH3.6*, and *CpGH3.11* expression increased significantly, while *CpGH3.9* expression decreased significantly. Expression of two other *CpGH3* genes, *CpGH3.1b* and *CpGH3.5*, peaked at 15 days, and then declined slightly at 20 and 25 days (Figure 4A). Next, we examined changes in IAA-amido synthetase activity using aspartate as the substrate for conjugation. A large increase (over 5-fold) in enzyme activity was detected during the postharvest process. IAA-Asp synthetase activity was induced significantly at 10 days, and reached its peak at 15 days (Figure 4B).

### Involvement of AsA in maintenance of shelf life of papaya fruit

**Figure 5:**
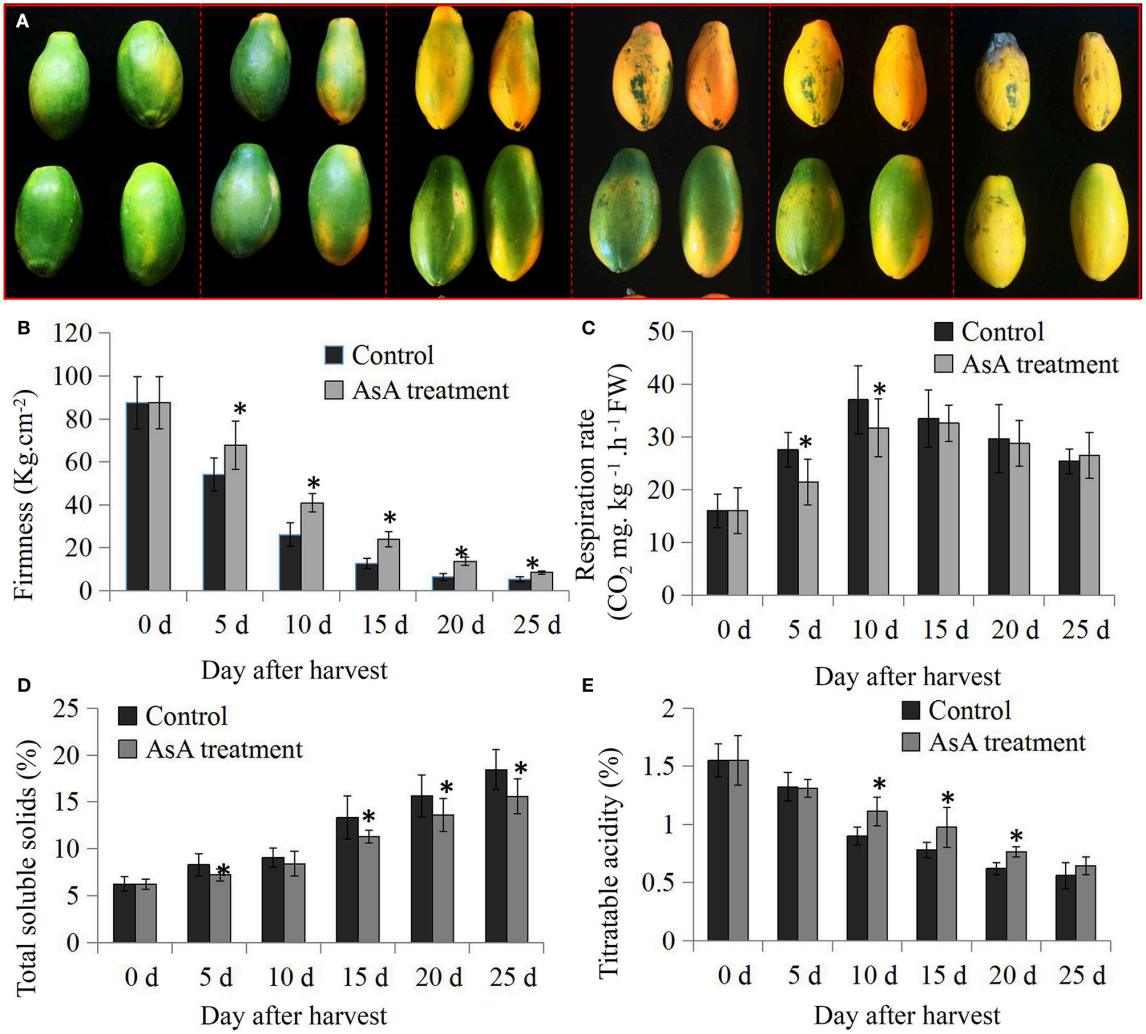
**Involvement of ascorbic acid (AsA) in maintenance of shelf life in papaya fruit. (A)** Morphological differences between control and AsA-treated papaya fruit during the postharvest period. The changes in firmness **(B)**, respiration rate **(C)**, total soluble solids **(D)**, and titratable acidity **(E)** between the control and AsA-treated fruit after a 25 day postharvest storage period. Significant differences (*P* < 0.05) between the control and AsA-treated fruit at different postharvest stages are indicated by an asterisk.

An enhanced AsA pool has been reported to be associated with good postharvest storage characteristics in various fruit species (Mellidou et al., 2012). However, the effects of AsA treatment on papaya fruit ripening are largely unknown. We found here that a 250 mM AsA treatment delayed the ripening process in papaya fruit (Figure 5A). The physiological data showed that the firmness of control fruit rapidly decreased and that 93.9% of their firmness was lost within 25 days after harvest. In contrast, the firmness of the AsA-treated fruit was slightly higher than the control fruit during postharvest storage (Figure 5B). The rate of CO_2_ production showed the characteristic respiratory climacteric pattern during postharvest storage for 25 days. In control fruit, the respiration rate lightly increased within 10 days after harvest, and then decreased slowly. In the AsA-treated fruit, the respiration rate was lower than in control fruit within 10 days after harvest. CO_2_ production peaked at 37.09 and 31.78 mg.kg^−1^ FW h^−1^ in the control and AsA-treated fruits, respectively, at 10 days (Figure 5C). Total soluble solids tended to increase during storage. However, the AsA treatment delayed the increase in total soluble solids compared to the controls (Figure 5D). Titratable acidities of the papaya fruit tended to decrease during postharvest storage. The AsA treatment delayed these decreases in titratable acidities compared with the control fruits, particularly between 10 and 20 days (Figure 5E).

### IAA-amido synthetase activities and the expression of *CpGH3* genes during postharvest after AsA treatment

**Figure 6:**
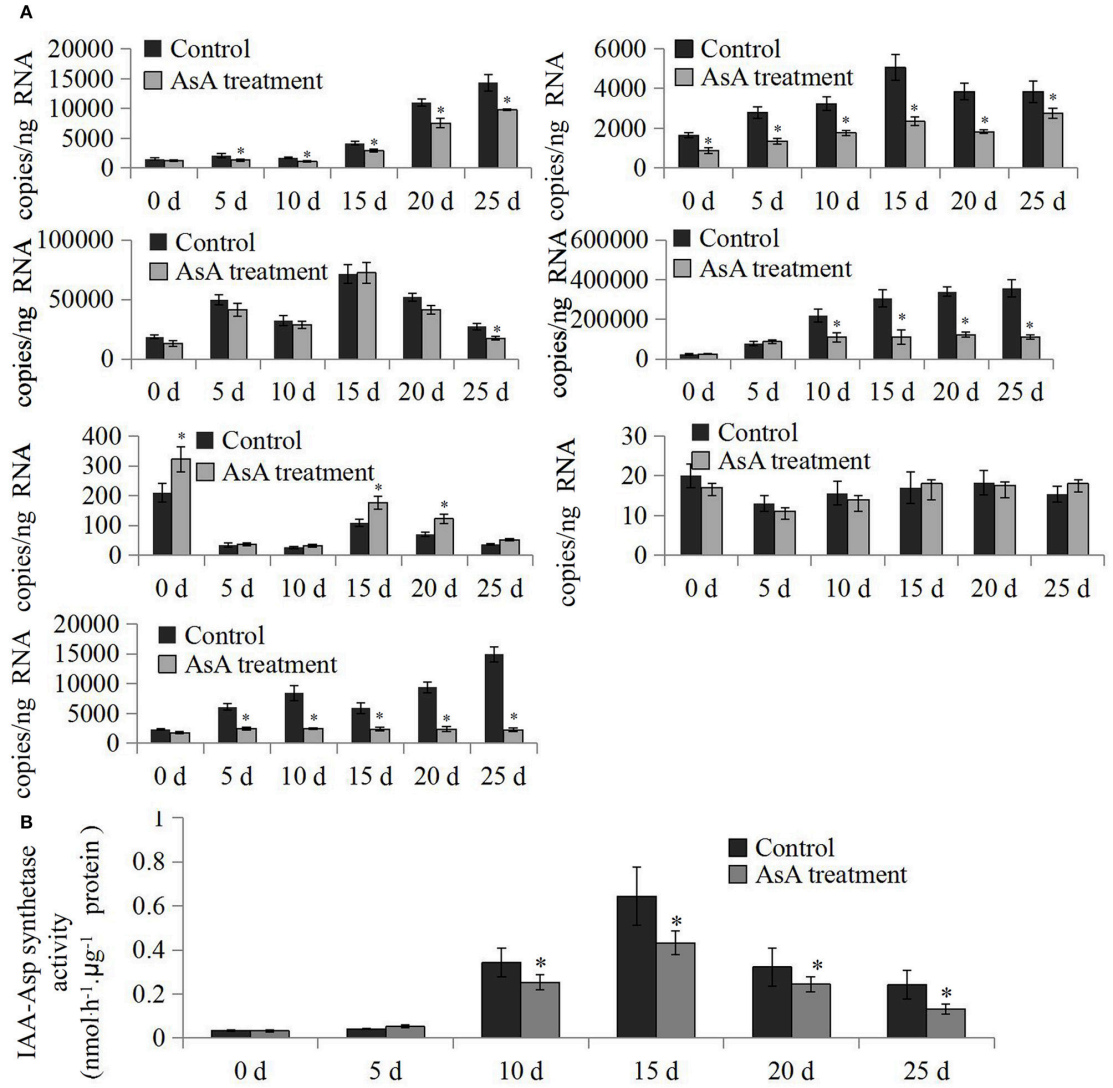
**Differences in *CpGH3* gene expression and IAA-amido synthetase activities between control and AsA–treated fruit. (A)** Analysis of expression of *CpGH3* family genes during different postharvest stages in control and AsA-treated fruit. **(B)** Enzymatic activities of IAA-amido synthetase. The data were obtained from five independent repeats, and standard deviations are shown with error bars. Significant differences (*P* < 0.05) between the control and AsA-treated fruit are indicated by an asterisk.

*CpGH3* gene expression and IAA-amido synthetase activities were measured during the postharvest period in both control and AsA-treated fruits. The expression of *CpGH3.1a, CpGH3.1b*, and *CpGH3.5* was largely reduced by the AsA treatment during different postharvest stages. In contrast, *CpGH3.6* showed a small increase at postharvest 0 and 5 days, and was then reduced at postharvest 10–25 days. Expression of *CpGH3.9* slightly increased after AsA treatment. *CpGH3.6* showed over 10-fold reduction in expression level after postharvest 15 days (Figure 6A). At postharvest stages 0 and 5 days, IAA-amido synthetase activities were similar in control and AsA treated fruit. During 10–25 days, AsA treatment significantly reduced IAA-amido synthetase activity compared to controls (Figure 6B).

### Endogenous IAA and ethylene production rate measurements

**Figure 7:**
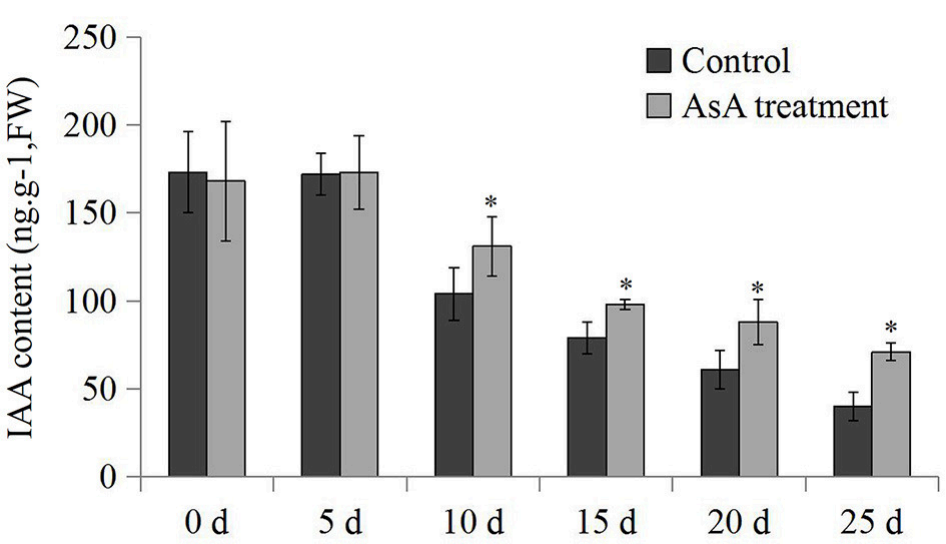
**The IAA content measurements**. The differences in IAA contents between the controls and AsA-treated fruits during the postharvest process. The data were analyzed by five independent repeats, and standard deviations were shown with error bars. The significant (*P* < 0.05) differences between the controls and AsA treatments are indicated by an asterisk.

To examine the role of endogenous IAA during storage, IAA contents were measured in papaya fruit under different conditions. Endogenous IAA contents fell during the postharvest period. Although IAA contents were consistent at 0 and 5 days, they then decreased significantly from 172 to 45 ng.g^−1^ FW (fresh weight). In contrast to the controls, the IAA contents of the AsA-treated fruit only decreased to 71 ng.g^−1^ FW (Figure 7). Thus, the AsA treatment may play a role in the retention of endogenous IAA during the postharvest period. Furthermore, previous studies have revealed that there is an ethylene-releasing peak during fruit ripening (Mo et al., 2008). In our study, production rate of ethylene climbed greatly with ripening and reached climacteric peak at the 10 days in the control, and then decreased gradually. In contrast to the control, the production rate of ethylene only increased 16.5 μL.kg^−1^.h^−1^, and reached its peak at the 15 days (Figure S6).

## Discussion

Papaya is a highly perishable fruit that undergoes a rapid softening process after harvest (Yao et al., 2014). Fruit ripening is associated with various hormone signals involved in the postharvest storage of fruits. GH3 proteins are among the most important downstream targets of auxin; GH3-mediated auxin homeostasis plays a vital part in the regulation of fruit ripening (Böttcher et al., 2010; Xie et al., 2015). Here, we performed a systematic identification of *CpCH3* genes, and analyzed their expression patterns and synthetase activities at different postharvest stages. The data from these analyses provide insights into the underlying mechanisms linking auxin and fruit ripening in papaya.

Here, seven *GH3* genes were identified in the papaya genome. This number is considerably smaller than the 19 genes identified in *A. thaliana* (Staswick et al., 2005). The relatively small size of the papaya genome (372 Mbp) may be a possible explanation for the comparatively small number of *CpGH3* genes (Ming et al., 2008). The similarities in characteristic motifs and exon-intron structures of the *CpGH3* genes with those reported *AtGH3* genes supported our identification. The presence of conserved domains in the CpGH3 proteins showed they were highly similar to GH3 proteins of other model plant species (Staswick et al., 2005; Jain et al., 2006). This similarity suggests that GH3 proteins might function in the same manner in different plant species. Furthermore, the phylogenetic analysis identified three orthologous gene pairs with high bootstrap values (100%), indicating a close relationship between the *A. thaliana* and papaya *GH3* gene families (Figure 1).

To identify potentially functional amino residues in the seven CpGH3 proteins, we performed a multiple sequence alignment using the sequences of the 19 AtGH3 proteins. We found that the CpGH3 proteins could be grouped into three subfamilies with different structures and different acyl acid substrate preferences (Wang et al., 2010). Recently, the crystal structures of two representative GH3 proteins, AtGH3.11 and AtGH3.12, were determined, and several specific secondary structures, such as the conserved motifs α5, α6, β8, and β9, were identified for acyl acid preferences (Westfall et al., 2012). Our data showed that five CpGH3 proteins, CpGH3.1a, CpGH3.1b, CpGH3.5, CpGH3.6, and CpGH3.9, were grouped into subfamily II, and might function as IAA-specific amido synthetases. Interestingly, the α5 motif was absent from CpGH3.9, although the α6, β8, and β9 motifs were present and residues 217 and 239 showed high similarity to the other subfamily II GH3 proteins. The acyl acid sites of IAA-using GH3 proteins have been reported to display consistent residues in the α5 motif (Westfall et al., 2012). Thus, the CpGH3.9 protein was grouped into subfamily II as an IAA-specific amido synthetase. Previous studies have identified the activities of several known GH3s in different plant species. In grape berry, the activity of the indole-3-acetic acid-amido synthetase GH3-1 has been identified (Böttcher et al., 2010). Moreover, GH3-2 was also identified as an IAA-amido synthetase with similar amino acid preferences as GH3-1 by the same group (Böttcher et al., 2011). In pea, the IAA-amide synthetase activity of PsGH3-5 was determined with aspartate as a substrate (Ostrowski and Jakubowska, 2013). In our study, five of the seven CpGH3 proteins showed IAA-specific amido synthetase activities, indicating their major roles in IAA-homeostasis.

Expression analyses suggest that *GH3* genes in different plant species have diverse roles in plant morphogenesis (Nakazawa et al., 2001; Takase et al., 2004; Khan and Stone, 2007; Kuang et al., 2011). Therefore, we analyzed the tissue-specific expression pattern of *CpGH3* genes to provide insights into their putative functions in papaya. *CpGH3.5* and *CpGH3.6* predominantly expressed in fruit, suggesting a possible role in auxin homeostasis during fruit development and ripening. Transcripts of *CpGH3.10* were virtually undetectable in fruit, indicating that this gene had limited or no role during postharvest stages of fruit development. In tomato, several *SlGH3* genes show different patterns of expression in reproductive tissues or fruit development stages. In particular, *SlGH3.1* and *SlGH3.2* exhibit ripening-associated expression patterns (Kumar et al., 2012). In papaya, expression of *CpGH3.1a, CpGH3.6*, and *CpGH3.11* exhibited a fruit softening-associated up-regulation; the remaining *CpGH3* genes showed a constant level of expression during different postharvest stages (Figure 4A). The differential expression of *CpGH3* genes in a stage-specific manner during fruit ripening and softening is a common characteristic of plant *GH3* genes (Böttcher et al., 2010; Kuang et al., 2011).

IAA is a well-studied inhibitor of ripening in both climacteric and non-climacteric fruits. A decrease in endogenous IAA levels is required for the initiation of ripening and has been reported to be a prerequisite for ripening to occur (Purgatto et al., 2002; Böttcher et al., 2010). It has been suggested that IAA-amido synthetase has an essential role in the ripening process through inactivation of endogenous IAA in pungent pepper and grape vine (Liu et al., 2005; Böttcher et al., 2010). We examined IAA-amido synthetase activities in papaya using aspartate as a substrate for conjugation during postharvest stages. A significant increase in enzyme activity was observed after postharvest stage 3 (Figure 4B), although expression of only three *CpGH3* genes was up-regulated. The increased expression of *CpGH3.1a, CpGH3.6*, and *CpGH3.11* suggest they might play a dominant role in the increase in enzyme activity during postharvest maturation. Interestingly, the mRNA levels of some *CpGH3* genes don't really correspond to enzymatic activity, suggesting the presence of diverse regulation manners in mRNA and protein levels. In fleshy fruit, the levels of endogenous IAA concentrations decline toward the onset of ripening (Buta and Spaulding, 1994). Many studies have reported that IAA levels are high at the early stages and then decrease to low levels at later ripening stages in non-climacteric fruit (Zhang et al., 2003; Symons et al., 2006). In *V. vinifera*, expression of *VvGH3.2* can be induced in pre-ripening berries by IAA treatment, and is associated with an increase in IAA-Asp levels and a decrease in free IAA levels (Böttcher et al., 2011). In common with these reports, IAA levels in papaya fruit were found to decline and to be relatively constant throughout the later stages of the postharvest period (Figure 7). Conjugation of IAA to amino acids is catalyzed by GH3 proteins, suggesting a negative feedback loop to regulate auxin homoeostasis (Staswick et al., 2005). The induced IAA-amido synthetase activities provide a possible explanation for the maintenance of low levels of endogenous IAA during the postharvest period in papaya fruit.

AsA is a well-known antioxidant that effectively regulates the enzymatic browning of fruits (Huang et al., 2014). The application of AsA is a useful approach to improve oxidative stress tolerance and to extend the shelf life of fruit (Zoldners et al., 2005; Liu et al., 2014). Several important postharvest physiological parameters, including fruit firmness, respiration rate, soluble solids content, and titratable acidity, were measured in the present study. Our analyses confirmed that AsA application delayed softening of papaya fruit (Figure 5). However, whether GH3 related IAA homoeostasis participated in this AsA-mediated effect is still largely unknown.

Analysis of *CpGH3* gene expression and enzymatic activities of CpGH3 proteins provided further insights into their possible functions during postharvest fruit storage. The qRT-PCR data showed that the expression of most *CpGH3* genes was decreased by the AsA treatment compared with the control, although *CpGH3.9, CpGH3.6*, and *CpGH3.10* showed evidence of a slight induction effect. Clear differences were observed among the *CpGH3* genes in their responses to AsA treatment suggesting variations in the transcriptional regulation of these genes (Böttcher et al., 2015). On the basis of gene expression levels, IAA-amido synthetase activities were reduced by AsA treatments from 10 to 25 days compared to controls. Our data suggested that AsA treatment regulated postharvest fruit ripening and softening by promoting endogenous IAA levels. Moreover, fruits treated with AsA showed a relatively lower production rate of ethylene compared to the controls. It suggested that AsA delayed ripening by regulating auxin-ethylene balance. The higher IAA levels in AsA treated fruits would lead to lower ethylene levels.

In this study, we identified seven *CpGH3* genes in a papaya genome database. Our study provides comprehensive information on *GH3* gene expression patterns in different tissues and on the enzyme activities of IAA-amido synthetases under different postharvest conditions. These results further indicated an important role for *GH3* genes in the regulation of auxin-associated fruit postharvest changes. Our findings may provide a way to develop novel strategies for improving papaya fruit quality during postharvest storage.

## Author contributions

KL, HL, and SF carried out the molecular studies. JZ and YP took care the plants. KL and JW drafted the manuscript. SF performed the statistical analysis. HL and SF conceived of the study, and participated in its design. CY acquired of funding and helped to draft the manuscript. All authors read and approved the final manuscript.

### Conflict of interest statement

The authors declare that the research was conducted in the absence of any commercial or financial relationships that could be construed as a potential conflict of interest.
