# Supplementary material for: Identification, Expression and IAA-Amide Synthetase Activity Analysis of Gretchen Hagen 3 in Papaya Fruit (Carica papaya L.) during Postharvest Process
Source: Front Plant Sci. 2016 Oct 20;7:1555. doi: 10.3389/fpls.2016.01555 (PMC5071377; doi:10.3389/fpls.2016.01555)
Supplement: Supplementary file 1 [file Table1.DOCX]

**Table S1:** The information of *GH3* family gene in *Arabidopsis*.

| Species | Gene | Name |
| --- | --- | --- |
| *Arabidopsis*^a^ | AT2G14960.1 | AtGH3.1 |
|  | AT4G37390.1 | AtGH3.2 |
|  | AT2G23170.1 | AtGH3.3 |
|  | AT1G59500.1 | AtGH3.4 |
|  | AT4G27260.1 | AtGH3.5 |
|  | AT5G54510.1 | AtGH3.6 |
|  | AT1G23160.1 | AtGH3.7 |
|  | AT5G51470.1 | AtGH3.8 |
|  | AT2G47750.1 | AtGH3.9 |
|  | AT4G03400.1 | AtGH3.10 |
|  | AT2G46370.1 | AtGH3.11 |
|  | AT5G13320.1 | AtGH3.12 |
|  | AT5G13350.1 | AtGH3.13 |
|  | AT5G13360.1 | AtGH3.14 |
|  | AT5G13370.1 | AtGH3.15 |
|  | AT5G13380.1 | AtGH3.16 |
|  | AT1G28130.1 | AtGH3.17 |
|  | AT1G48660.1 | AtGH3.18 |
|  | AT1G48670.1 | AtGH3.19 |
|  |  |  |

a. The information of *Arabidopsis* *GH3* genes was from <http://www.arabidopsis.org/>.
