# Supplementary material for: Identification, Expression and IAA-Amide Synthetase Activity Analysis of Gretchen Hagen 3 in Papaya Fruit (Carica papaya L.) during Postharvest Process
Source: Front Plant Sci. 2016 Oct 20;7:1555. doi: 10.3389/fpls.2016.01555 (PMC5071377; doi:10.3389/fpls.2016.01555)
Supplement: Supplementary file 3 [file Table3.DOCX]

**Table S3:** The information of *GH3* family genes in *C. papaya*.
