# Supplementary material for: Identification, Expression and IAA-Amide Synthetase Activity Analysis of Gretchen Hagen 3 in Papaya Fruit (Carica papaya L.) during Postharvest Process
Source: Front Plant Sci. 2016 Oct 20;7:1555. doi: 10.3389/fpls.2016.01555 (PMC5071377; doi:10.3389/fpls.2016.01555)
Supplement: Supplementary file 4 [file Table4.DOCX]

**Table S4:** Numbers of stress-related *cis*-elements in the upstream 1.5 kb regions of *CpGH3* family genes
