# Supplementary figures and images for: Identification, Expression and IAA-Amide Synthetase Activity Analysis of Gretchen Hagen 3 in Papaya Fruit (Carica papaya L.) during Postharvest Process
Source: Front Plant Sci. 2016 Oct 20;7:1555. doi: 10.3389/fpls.2016.01555 (PMC5071377; doi:10.3389/fpls.2016.01555)

**Fig.S1:** The standard curves for absolute quantification RT-PCR of *CpGH3* genes.

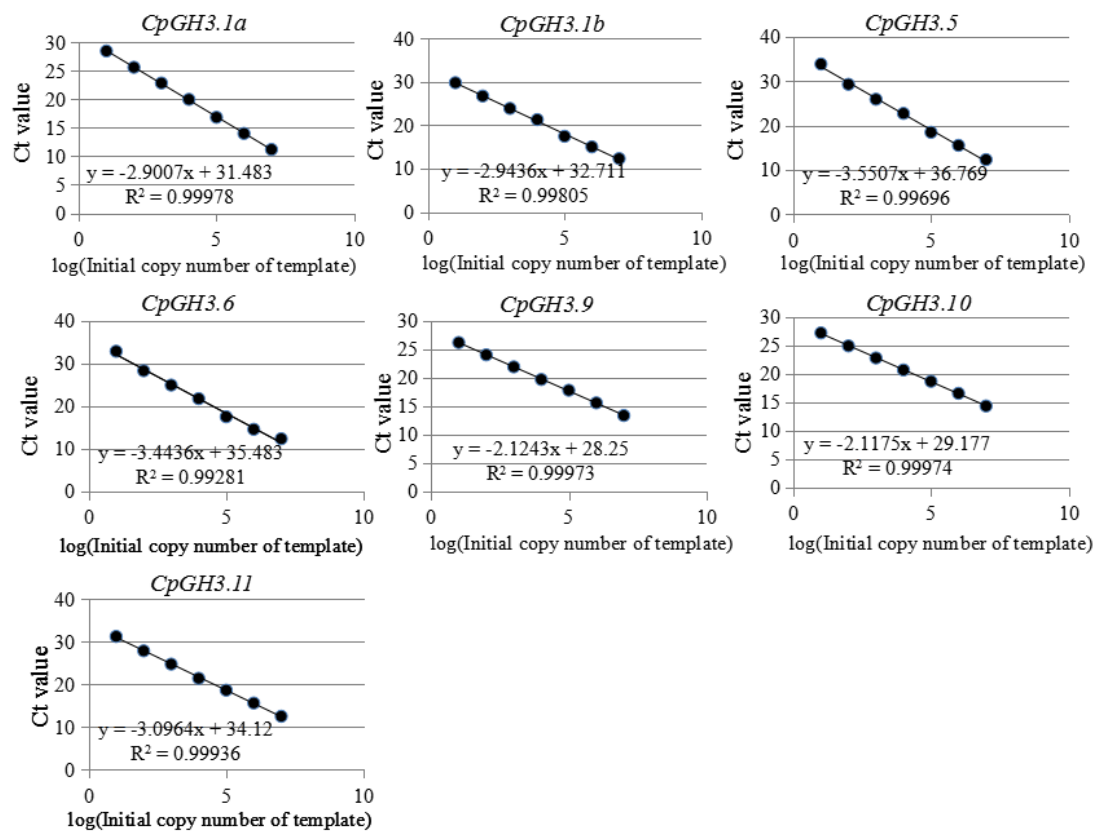

Supplement: Supplementary file 5 [file Image1.PDF]
