# Supplementary material for: Identification, Expression and IAA-Amide Synthetase Activity Analysis of Gretchen Hagen 3 in Papaya Fruit (Carica papaya L.) during Postharvest Process
Source: Front Plant Sci. 2016 Oct 20;7:1555. doi: 10.3389/fpls.2016.01555 (PMC5071377; doi:10.3389/fpls.2016.01555)
Supplement: Supplementary file 6 [file Image2.PDF]

Fig. S2: Exon-intron structure analysis of CpGH3 genes.

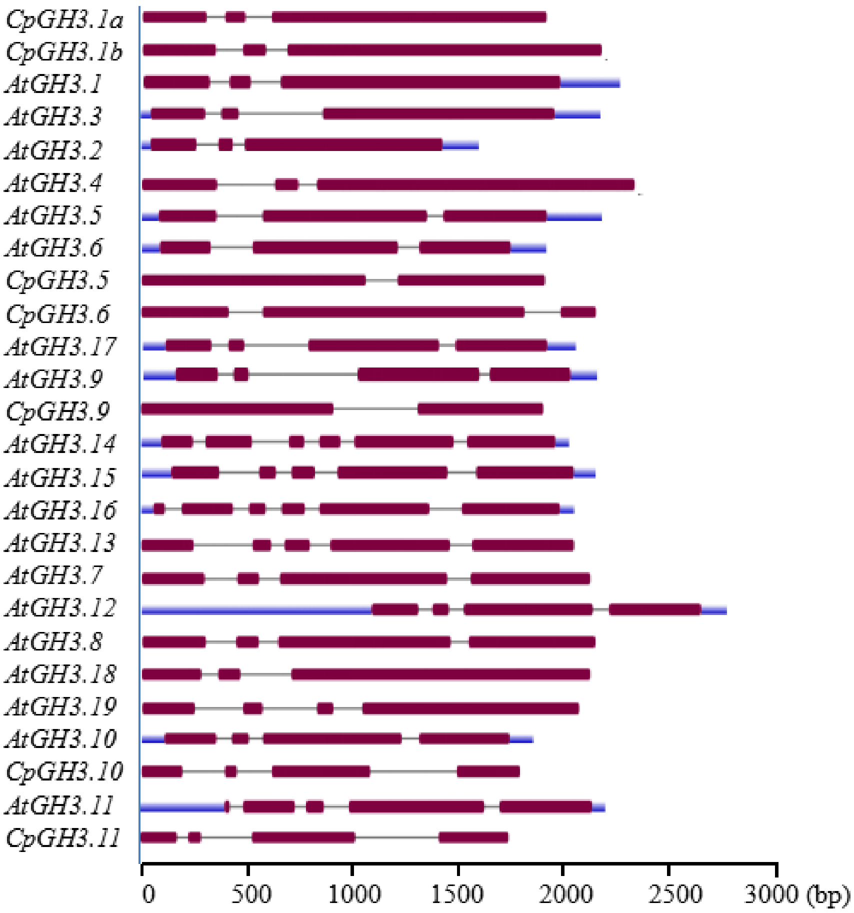

Fig.S2 **Exon-intron structure analysis of CpGH3 genes.** Exon-intron structure analysis of CpGH3 genes is showed to the right of the figure. The untranslated regions (UTRs) are indicated by thick blue lines; the exons are indicated by red boxes; introns are indicated by gray lines.
