# Supplementary material for: Identification, Expression and IAA-Amide Synthetase Activity Analysis of Gretchen Hagen 3 in Papaya Fruit (Carica papaya L.) during Postharvest Process
Source: Front Plant Sci. 2016 Oct 20;7:1555. doi: 10.3389/fpls.2016.01555 (PMC5071377; doi:10.3389/fpls.2016.01555)
Supplement: Supplementary file 7 [file Image3.PDF]

Fig. S3: The multiple sequence alignments of GH3 proteins from papaya and *Arabidopsis*.

```
AtGH3.16    MTPFICTERERDGSDPIHCSQRKDNRKAAIMLPK--FDPTNPLATMSVLEDVTTNVKKIQ
AtGH3.13    ------------------------------MLPK--FDLTDPKASLSLLEDVTTNVTQIQ
AtGH3.14    ------------------------------MLPK--FDPTDQKACLSLLEDVTTNVKQIQ
AtGH3.15    ------------------------------MLPK--FDPTNQKACLSLLEDLTTNVKQIQ
AtGH3.7     -------------------MSL----------TS--DLSEKSSDKMKVLEDLTSNVTQIQ
AtGH3.12    -------------------MK---------------PIFDINETFEKQLKDLTSNVKSIQ
AtGH3.8     -------------------MSL----------CS--DLTE--KLDEEILEDLTSNVKQIQ
AtGH3.18    -------------------------------------MSL--SVELKDLEVLTTNAKQIQ
AtGH3.19    ------------------------------------MMNP--SLNLMDLEELTSNAKQIQ
CpGH3.6     -------------------MPEAPKTNSY-------NLSEKNKKMLQFIEDVTINADEVQ
CpGH3.5     ------------------------------------------------------------
AtGH3.5     -------------------MPEAPKKESLEVFDL--TLDQKNKQKLQLIEELTSNADQVQ
AtGH3.6     -------------------MPEAPKIAALEVSDE--SLAEKNKNKLQFIEDVTTNADDVQ
AtGH3.3     -------------------MTVDS-ALRSPMMH---SPSTKDVKALRFIEEMTRNVDFVQ
AtGH3.2     -------------------MAVDS-PLQSRMVSA--TTSEKDVKALKFIEEMTRNPDSVQ
AtGH3.4     -------------------MAVDS-LLQSGMASP--TTSETEVKALKFIEEITRNPDSVQ
AtGH3.1     -------------------MAVDS-NLSSPLGP---PACEKDAKALRFIEEMTRNADTVQ
CpGH3.1a    -------------------MAVDS-AISSPLGP---PACEKDAKALQFIEEMTRNANLVQ
CpCH3.1b    -------------------MAVDS-ALSSPLGP---PACERDAKALQFIDEMTRNTDAVQ
AtGH3.17    ------------------------------MIPS--YDPNDTEAGLKLLEDLTTNAEAIQ
AtGH3.9     --------------------------------MD--VMKLDHDSVLKELERITSKAAEVQ
CpGH3.9     ------------------------------------------------------------
AtGH3.10    ----------------------------------METVEAGHDDVIGWFEHVSENACKVQ
CpGH3.10    ----------------------------MELGRGVSNGNHGDGDIIRWFEEVTENAGEVQ
AtGH3.11    ------------------------------MLEKV--ETFDMNRVIDEFDEMTRNAHQVQ
CpGH3.11    ------------------------------MLEKM--ESFDVNREIEEFEAVTKDAERVQ
```

```
AtGH3.16    DSLLEAILSRNSQTEYLRGF-LTGQ------------LD--KQSFKKNVPIVTYEDIKPH
AtGH3.13    DSILEAVLSRNAHTEYLKGF-LNGQ------------VD--KQTFKKNVPIVTYEDIKPY
AtGH3.14    DSVLEAILSRNAHTEYLSGF-LNGQ------------AD--KKSFKKNVPVVTYEDIKPY
AtGH3.15    DSVLEAILSRNAQTEYLRGF-LNGQ------------VD--KQNFKKNVPVVTYEDIRSY
AtGH3.7     DNVLEEILTLNANTNYLQKF-FLGS------------FD--KESFKKNVPVVTYEDVKPY
AtGH3.12    DNLLEEIITPNTKTEYLQRF-LIDR------------FD--KELFKKNVPIVSYEDIKPY
AtGH3.8     DNVLEEILTLNANTEYLRRF-LHGS------------SS--KELFKKNVPVVTYEDVKPF
AtGH3.18    DDVLKEILTLNANTEYLKRF-LDGS------------SD--KELFKKNVPVVSYNDVKPY
AtGH3.19    EDVLEEILTLNANTEYLHRF-LHGS------------SD--KVLFKKNVPVVTYDDVKPY
CpGH3.6     KKVLEEILSRNAHVEYLRKQGISGQ------------TD--RETFKKLVPVITYEDVQPY
CpGH3.5     ------------------------------------------------------------
AtGH3.5     RQVLEEILTRNADVEYLRRHDLNGR------------TD--RETFKNIMPVITYEDIEPE
AtGH3.6     RRVLEEILSRNADVEYLKRHGLEGR------------TD--RETFKHIMPVVTYEDIQPE
AtGH3.3     KKVIREILSRNSDTEYLKRFGLKGF------------TD--RKTFKTKVPVVIYDDLKPE
AtGH3.2     EKVLGEILTRNSNTEYLKRFDLDGV------------VD--RKTFKSKVPVVTYEDLKPE
AtGH3.4     EKVLGEILSRNSNTEYLKRFDLNGA------------VD--RKSFKSKVPVVIYEDLKTD
AtGH3.1     ENLLAEILARNADTEYLRRFNLCGA------------TD--RDTFKTKIPVITYEDLQPE
CpGH3.1a    EKVLAEILSRNAETEYLKRFKLNGA------------ID--RDTFKSKLPVITYEDLQPE
CpCH3.1b    ERVLCEILSRNSETEYLRRFHLNGA------------TD--RDTFKSKVPVVTYEDLQPD
AtGH3.17    QQVLHQILSQNSGTQYLRAF-LDGE------------ADKNQQSFKNKVPVVNYDDVKPF
AtGH3.9     DNILRGILERNKDTEYLSKY-MNGS------------KD--VLEFKRAVPIIIYKDIYPY
CpGH3.9     ------------------------------------------------------------
AtGH3.10    SETLRRILELNSGVEYLRKWLGTVDVE-------KMDDYTLETLFTSLVPIVSHADLDPY
CpGH3.10    RETLGRILKENYGVEYLKKWIGEEEEEEMVKRIQEMDINALESLYTSLVPLASHGDLEPF
AtGH3.11    KQTLKEILLKNQSAIYLQNCGLNGN------------ATDPEEAFKSMVPLVTDVELEPY
CpGH3.11    RETLKKILEENGSAEYLQNLGLNGR------------TD--PETFKAFVPIVTHNELEPY
```

```
AtGH3.16      IDRIANGEPSDLICDRPISLLLATTGTSGGIPKLIPLTAEELEQRILFGFLYVPLVFKHI
AtGH3.13      INRIANGEASDLICDRPISLLVMSSGTTAGIQNLIPLTTEDGEQRIMFGSLYRSLLYKYV
AtGH3.14      IDRIANGEPSDLICDRPISVLLTSSGTSGGVPKLIPLTTEELEQRISFASLYRPLLYKYI
AtGH3.15      IDRIANGEPSDLICDRPISVLLTSSGTSGGVPKLIPLTTEDLEQRISFSSLYAPLLYKHI
AtGH3.7       IERVVNGEPSNVISARPITGFVLSTGTSGGAQKMMPWNEKYLDNLTFMYDLRMHIISNNV
AtGH3.12      LDRVVNGESSDVISARTITGFLLSSGTSGGAQKMMPWNNKYLDNLTFIYDLRMQVITKHV
AtGH3.8       IDRVTNGEPSDIISGNPITGFLLSSGTSGGKQKMFPRNNKYLENIKFIFYYRSLVISKHI
AtGH3.18      IERVANGEPSDVISGGTITRFVQSTGTSGGIHKIFPVNDKYIENLGYLLAVSSLITSN--
AtGH3.19      IERVANGEPSDVISGGPITMFLRSTGTSGGKQKVFPVNDKYIEKLGYVIALRSLAMSKHF
CpGH3.6       INRIANGDTSPILCSKPISEFLTSSGTSGGERKLMPTIEEELDRRSLLYSLLMPVMSQYV
CpGH3.5       ----------------------------------MPTIEEELDRRSLLYSLLMPVMSQYV
AtGH3.5       INRIANGDKSPILSSKPISEFLTSSGTSGGERKLMPTIEEELDRRSLLYSLLMPVMSQFV
AtGH3.6       INRIANGDKSQVLCSNPISEFLTSSGTSGGERKLMPTIEEELDRRSLLYSLLMPVMDQFV
AtGH3.3       IQRIANGDRSMILSSYPITEFLTSSGTSAGERKLMPTIDEDMDRRQLLYSLLMPVMNLYV
AtGH3.2       IQRISNGDCSPILSSHPITEFLTSSGTSAGERKLMPTIEEDLDRRQLLYSLLMPVMNLYV
AtGH3.4       IQRISNGDRSPILSSHPITEFLTSSGTSAGERKLMPTIEEDINRRQLLGNLLMPVMNLYV
AtGH3.1       IQRIADGDRSPILSAHPISEFLTSSGTSAGERKLMPTIKEELDRRQLLYSLLMPVMNLYV
CpGH3.1a      IQRIASGDRSAILSAHPISEFLTSSGTSAGERKLMPTIQEELDRRQLLYSLLMPVMNLYV
CpCH3.1b      IQRIANGDRSAIFSSYPISEFLTSSGTSAGERKLMPTIHEELDRRCLLYSLLMPVMNLYV
AtGH3.17      IQRIADGESSDIVSAQPITELLTSSGTSAGKPKLMPSTAEELERKTFFYSMLVPIMNKYV
AtGH3.9       IQRIANGEDSSLITGHSITEILCSSGTSAGEPKLMPTIPEDLDRRTFLYNLIIPIVNKYI
CpGH3.9       ------------------------------------------------------------
AtGH3.10      IQRIADGETSPLLTQEPITVLSLSSGTTEGRQKYVPFTRHSAQTTLQIFRLSAAYRSRFY
CpGH3.10      IQRIADGDTSPLLTSQPIQNLSLSSGTTEGRQKYVPFTRHSSQTTLQVFKLAAAYRSRAY
AtGH3.11      IKRMVDGDTSPILTGHPVPAISLSSGTSQGRPKFIPFTDELMENTLQLFRTAFAFRNRDF
CpGH3.11      IQRIADGDSSPILTGKRITTISLSSGTTQGRQKFVPFNDDLMDNTMQIYRTSFAFRNREF


AtGH3.16      EG-LTQGKSLMFYFVTRESETVSGLMVRFMITCVLKSVNP-TNSFLWDRVQISPHAIAIC
AtGH3.13      EG-IREGKSLTFYFVNPERETASGILIRTMITCILKSVNK-TNSSLWDRLQISPHEISTC
AtGH3.14      EG-IRERKSFMLYFVTRESETASGILVRTMITCVLKSVTP-ANSFIWDQSQISPHAITTC
AtGH3.15      DG-LSEGKSLIFYFVTRESKTANGLMVRTMVTSFLKSIKQ-TNSFLWDSLQVSPHAITTC
AtGH3.7       KD-VEKGKAMMFYFTKLESITPSGLPARVASSSYLKSNYFKNRPSNWYYSYTSPDEVTLC
AtGH3.12      KG-VEEGKGMMFLFTKQESMTPSGLPARVATSSYFKSDYFKNRPSNWYYSYTSPDEVILC
AtGH3.8       DG-LEHGKGMVFNFCTSENTTPSGLPSSAASTSFFKSDYFKNRPSYWHWSYTSPDEVILC
AtGH3.18      DKVDEKGKKMAFLYNRLESKTPSGLALSSSFTSYFMSDYFKNRSSKCNSEYTSPDQVILC
AtGH3.19      DSGGEQGKAMEFHCTKPPSATPSGL-----------------------------------
CpGH3.6       PG-LEKGKGMYFYFIKSEAKTPGGLLARPVLTSYYKSSHFKDRPYDPYTNYTSPNETILC
CpGH3.5       PG-LEKGKGMYFYFIKSEAKTPGGLLARPVLTSYYKSSHFKDRPYDPYTNYTSPNETILC
AtGH3.5       PG-LENGKGMYFLFIKSESKTPGGLPARPVLTSYYKSSHFKERPYDPYTNYTSPNETILC
AtGH3.6       PG-LDKGKGMYFLFIKSESKTPGGLPARPVLTSYYKSSHFKNRPYDPYTNYTSPNQTILC
AtGH3.3       PG-LDKGKALYFLFVKTESKTPGGLPARPVLTSYYKSEQFKRRPNDPYNVYTSPNEAILC
AtGH3.2       PG-LDKGKGLYFLFVKSESKTSGGLPARPVLTSYYKSDHFKRRPYDPYNVYTSPNEAILC
AtGH3.4       PG-LDKGKGLYFLFVKSESTTSGGLPARPALTSYYKSDYFRTS--DSDSVYTSPKEAILC
AtGH3.1       PG-LDKGKGMYFLFVKSETKTPGGLPARPVLTSYYKSEHFRSRPYDPYNVYTSPNEAILC
CpGH3.1a      PG-LDKGKGLYFLFVKSETRTPGGLLARPVLTSYYKSDHFKTRPYDPYSVYTSPNEAILC
CpCH3.1b      PG-LDQGKGLYFLFVKAETRTPGGLLARPVLTSYYKSEHFKARPYDPYNVYTSPNEAILC
AtGH3.17      DG-LDEGKGMYLLFIKPEIKTPSGLMARPVLTSYYKSQHFRNRPFNKYNVYTSPDQTILC
AtGH3.9       TG-LDKGKAMYLNFVKAETSTPCGLPIRAVLTSYYKSKHFQCRPYDPFNDLTSPIQTILC
CpGH3.9       ------------------MSTPCGLPARAVLTSYYKSTHFKCRTSDPFNDFTSPDKAILC
AtGH3.10      PI-REGGRILEFIYAGKEFKTLGGLTVGTATTHYYASEEFKTKQETTKSFTCSPQEVISG
CpGH3.10      PI-REGGRILEFIYSSKQFKTKGGLTSGTATTHYYSSEEFKIKQEQTKAFTCSPEAVISG
AtGH3.11      PI-DDNGKALQFIFSSKQYISTGGVPVGTATTNVYRNPNFKAGMKSITSPSCSPDEVIFS
CpGH3.11      PI-G-TGKALQFVYSSKQFKTKGGLAAGTATTNVYRNPQFKSGMKAIMFQGCSPDEVIFG
                                 .    *.
```

```
AtGH3.16        EDTNQGMYCQLLCGLLQREHVARLGAPYASSFLKVIKFLEDHWPELCSNIRTGRLSDWIT
AtGH3.13        EDTTQSMYCQLLCGLLQRDNVARLGAPFASVFIRVIKYLEGHWQELCSNIRTGRLSDWIT
AtGH3.14        SDTTQSMYCQLLCGLLQRDNVGRLGAPFASSFLKIIKFLEDHWPEFCSNIRTGCLSDWIT
AtGH3.15        ADTTQSMYCQLLCGLLERDNVARLGAPFASSFLKVIKFLEDHWPELCSNIRTGRLSDWIT
AtGH3.7         PDNKQNLYCHLLCGLVQRNEVTRMGSIFASVMVRAIKFLEDSWEELCSNIRSGQLSEWIT
AtGH3.12        PNNTESLYCHLLCGLVQRDEVVRTGSIFASVMVRAIEVLKNSWEELCSNIRSGHLSNWVT
AtGH3.8         SDTKQTLYCHLLCGLVQRDDVVKVGAAFVTILVRAINLLENSWKEICTNIRFGHLSEWIT
AtGH3.18        PDNNQSVYCHLLCGLSQREKVVGVSATFAHALIKAINALQIYWKELSSNIRSGHVSEWIT
AtGH3.19        ----------------PRDEVVRVGAAFAFVLVRAIDFLEKHWKELCSNIRSGHVSEWIT
CpGH3.6         PDSYQSMYSQLLCGLCLHKEVLRVGAVFASGFIRAIRFLEKHWPLLCNDIRTGVLDAQIT
CpGH3.5         PDSYQSMYSQLLCGLCLHKEVLRVGAVFASGFIRAIRFLEKHWPLLCNDIRTGVLDAQIT
AtGH3.5         SDSYQSMYSQMLCGLCQHQEVLRVGAVFASGFIRAIKFLEKHWIELVRDIRTGTLSSLIT
AtGH3.6         SDSYQSMYSQMLCGLCQHKEVLRVGAVFASGFIRAIKFLEKHWPELARDIRTGTLSSEIT
AtGH3.3         PDSSQSMYTQMLCGLLMRHEVLRLGAVFASGLLRAIGFLQTNWKELADDISTGTLSSRIS
AtGH3.2         SDSSQSMYAQMLCGLLMRHEVLRLGAVFASGLLRAISFLQNNWKELARDISTGTLSSRIF
AtGH3.4         CDSSQSMYTQMLCGLLMRHEVNRLGAVFPSGLLRAISFLQNNWKELSQDISTGTLSSKIF
AtGH3.1         PDSFQSMYTQMLCGLLDRLSVLRVGAVFASGLLRAIRFLQLHWSRFAHDIELGCLDSEIT
CpGH3.1a        ADSFQSMYTQMLCGLLERHHVLRLGAVFASGLLRAIKFLQLNWQELANDIKTGKLSQKIT
CpCH3.1b        ADSFQSMYTQMLCGLLMRQEVLRVGAVFASGLLRAIHFLQQNWKDLAHDISTGTLSSKIT
AtGH3.17        QDSKQSMYCQLLCGLVQRSHVLRVGAVFASAFLRAVKFLEDHYKELCADIRTGTVTSWIT
AtGH3.9         EDSNQSMYCQLLAGLIHRHKVMRLGAVFASAFLRAISYLEKKWSQLCEDIRTGSLNPMIT
CpGH3.9         NDSNQSMYCQLLAGLIHRHQVMRLGAVFASAFLRAISFLERKWMQLCHDIRTGQLDLMIT
AtGH3.10        GDFGQCTYCHLLLGLHYSSQVEFVASAFSYTIVQAFSFFEEIWREICADIKEGNLSSRIT
CpGH3.10        GDYKQSTYCHLLLGLYFSHQVEFITSTFAYSIVQAFSTFEDLWREICSDLKTGSLSSRIT
AtGH3.11        PDVHQALYCHLLSGILFRDQVQYVFAVFAHGLVHAFRTFEQVWEEIVTDIKDGVLSNRIT
CpGH3.11        PDFYQSLYCHLLCGLLFRNEVQFVFSTFAHSIVYSFRTFEQVWEELCTDIRDGVLSSRVS
                                    *    : :   ::  .  ::  :  :  ::  * :   :

AtGH3.16        DAQCVSGIGNFLTAPDPDLANLIEQECS---KT-SWEAILSRIWPKAKCIEAVITGTMAQ
AtGH3.13        DPQCVSGISKFLTAPNPDLASLIEQECS---KT-SWEAIVKRLWPKAKCVEAVVTGSMAQ
AtGH3.14        DPQCVSGIGKFLTAPNPELASLIEQECS---QT-SWEAIVKRLWPKAKCIEAIVTGTMAQ
AtGH3.15        DATCTSGIGKFLTAPNPELASLIEQECS---KT-SWEAILKRLWPKAKCIESIITGTMAQ
AtGH3.7         DIGCRDSVSLVLGGPHPEAADTIEQICN---QK-CWKGIITRLWPKAKYIETIVTGSMVQ
AtGH3.12        DLGCQNSVSLVLGGPRPELADTIEEICN---QN-SWKGIVKRLWPNTKYIETVVTGSMGQ
AtGH3.8         DISCRDSVSKILGEPNPELADLIENECN---NK-SWEGIVPRLWPKAKFIECIATGQMAQ
AtGH3.18        DLDCKNAVSAILGGPDPELADVIEQECS---HK-SWEGIITRLWPKAKFIECIVTGQMAQ
AtGH3.19        DLEGRNAVSTILRGPDSILADVIEQECS---HK-SWEGIITRLWPKAKYIDCIITGQMSQ
CpGH3.6         DQSVRESVMKIL-KPDPQLADFIEGECR---KE-SWQGIITKLWPNTKYIEVIVTGAMSQ
CpGH3.5         DQSVRESVMKIL-KPDPQLADFIEGECR---KE-SWQGIITKLWPNTKYIEVIVTGAMSQ
AtGH3.5         DPSVREAVAKIL-KPSPKLADFVEFECK---KS-SWQGIITRLWPNTKYVDVIVTGTMSQ
AtGH3.6         DSSVREAVGEIL-KPDPKLADFVESECR---KT-SWQGIITRLWPNTKYVDVIVTGTMSQ
AtGH3.3         DPAIKESMSKILTKPDQELADFITSVCG---QDNSWEGIITKIWPNTKYLDVIVTGAMAQ
AtGH3.2         DPAIKNRMSKILTKPDQELAEFLVGVCS---QE-NWEGIITKIWPNTKYLDVIVTGAMAQ
AtGH3.4         DHAIKTRMSNILNKPDQELAEFLIGVCS---QE-NWEGIITKIWPNTKYLDVIVTGAMAE
AtGH3.1         DPSIRQCMSGIL-KPDPVLAEFIRRECK---SD-NWEKIITRIWPNTKYLDVIVTGAMAQ
CpGH3.1a        DSSIRDCMAKILVKPDPELAGFVRAECA---TG-NWEGIITRIWPNTKYLDVIVTGAMAQ
CpCH3.1b        DPSIKECMSKIL-KPKPELAEFIAKECS---EE-NWEGIITRVWPNTKYLDVIVTGAMAQ
AtGH3.17        DSSCRDSVLSILNGPNQELADEIESECA---EK-SWEGILRRIWPKAKYVEVIVTGSMAQ
AtGH3.9         DPGCQMAMSCLLMSPNPELASEIEEICG---RS-SWKGILCQLWPKAKFIEAVVTGSMAQ
CpGH3.9         DPGCRSSMSALLVSPDQCLANEIEDICS---RA-SWRGILCLLWPKAKYIDAVVTGSMAQ
AtGH3.10        LPKMRKAVLALI-RPNPSLASHIEEICLELETNLGWFGLISKLWPNAKFISSIMTGSMLP
CpGH3.10        ILKMRKAVLEII-KPNPRLAMEIEEKLE--MGNGDWFGLIPKLWPNVKYVYSIMTGSMQP
AtGH3.11        VPSVRTAMSKLL-TPNPELAETIRTKCM---SLSNWYGLIPALFPNAKYVYGIMTGSMEP
CpGH3.11        VPSIRAAMSKLL-KPNPELADLIHTKCS---GLSNWYGLIPELFPNAKYVYGIMTGSMEP
                       :  .:  *    *  :            *  ::  ::*:.* :  : ** *
```

```
AtGH3.16    YIPLLEFYGGG-LPLVSSWYGSSECFIGINLNPLSKPSDVSYTIIPSMGYFEFIEVVKDR
AtGH3.13    YIPLLEFYGGG-LPLISSWYGSSECFMGVNVNPLCKPSDVSYTIIPSMAYFEFLEVKKDQ
AtGH3.14    YNPLLEFYSGG-LPVISTFYGSSECFFGLNLNPLSKPNEVSYTIIPCMAYFEFLEVEKDY
AtGH3.15    YIPLLEFYSGG-LPLTSSFYGSSECFMGVNFNPLCKPSDVSYTIIPCMGYFEFLEVEKDH
AtGH3.7     YVPTLNYYSNNMLPLISTIYASSETQFGLNLNPMCKPEDVSYTFMPNVSYFEFIPVDG--
AtGH3.12    YVPMLNYYCND-LPLVSTTYGSSETTFGINLDPLCKPEDVSYTFMPNMSYFEFIPMDG-G
AtGH3.8     HIPTLEFYSNK-LPSISSSYVSSETMFGINMSPLCKPENVSYTFLPNLSYFEFLLVDA-G
AtGH3.18    YIPTLDFYSNK-LPIVSMVYGSSESIFGVNVDPLSKPQDVSYTFLPNISYFEFLPIDHEE
AtGH3.19    YIPMLEFYSNK-LPIVSTTYGSSESTFGMNVDPLCKPQDTSYTCAPNISYFEFLPVDHKG
CpGH3.6     YIPTLDYYSNG-LPLVCTMYASSECYFGVNLKPMCKPSEVAYTLIPTMAYFEFLPVQRNN
CpGH3.5     YIPTLDYYSNG-LPLVCTMYASSECYFGVNLKPMCKPSEVAYTLIPTMAYFEFLPVQRNN
AtGH3.5     YIPTLDYYSNG-LPLVCTMYASSECYFGVNLRPLCKPSEVSYTLIPSMAYFEFLPVHRNN
AtGH3.6     YIPTLDYYSNG-LPLVCTMYASSECYFGVNLRPLCKPSEVSYTLIPNMAYFEFLPVHRNS
AtGH3.3     YIPMLEYYSGG-LPMACTMYASSESYFGINLKPMCKPSEVSYTIMPNMAYFEFLPHHEVP
AtGH3.2     YIPTLEYYSGG-LPMACTMYASSESYFGINLKPMCKPSEVSYTIMPNMAYFEFLPHNHDG
AtGH3.4     YIPMLEYYSGG-LPMASMIYASSESYFGINLNPMCKPSEVSYTIFPNMAYFEFLPHNHDG
AtGH3.1     YIPTLEYYSGG-LPMACTMYASSECYFGLNLNPMSKPSEVSYTIMPNMAYFEFIPLGG--
CpGH3.1a    YIPTLDYYSGG-LPLACTMYASSECYFGLNLNPMCKPSEVSYTIMPNMAYFEFLPHSPNS
CpCH3.1b    YIPTLEYYSGD-LPMTCTMYASSECYFGLNLKPMCKPSEVSYTIMPNMGYFEFLPHDPSS
AtGH3.17    YIPTLEFYSGG-LPLVSTMYASSECYFGINLNPLCDPADVSYTLLPNMAYFEFLPVDDKS
AtGH3.9     YIPALEFFSQGKIPLVCPMYASSETYFGVNVEPLSKPSDVVFTLLPNMCYFEFIPLGKNG
CpGH3.9     YIPSLEYYSGGKLPLVCTMYASSECYFGLNLKPLCDPADVSFTLLPNMGYFEFLPLGKNG
AtGH3.10    YLNKLRHYAGG-LPLVSADYGSTESWIGVNVDPHLPPEDVSFAVIPTFSYFEFIPLYRRQ
CpGH3.10    YAKKLRYYAGK-IPLVGADYGSTESWIGVNVDPCLPPEKVTFAVIPTFSYFEFIPLYRPE
AtGH3.11    YVPKLRHYAGD-LPLVSHDYGSSEGWIAANVTPRLSPEEATFAVIPNLGYFEFLPVSETG
CpGH3.11    YLKKLRHYAGK-LPLVCHDYGSSEGWIAANVNPKLPPESATFAVIPNIGYFEFIPLRHNA
            :   * .:    :*     * *:*  :. *. *   * .. ::  * . ****:

AtGH3.16    QEAG-----------HVPA-DPVVVDLVDVKIGHDYELLVTTFSGLYRYRLGDVLRVTGF
AtGH3.13    QEAG-----------LDPIENHVVVDLVDVKIGHDYEPVVTTFSGLYRYRVGDLLRVTGF
AtGH3.14    -ESG-----------HDPAENPVVVDLVDVKIGHDYEPVVTTFAGLYRYRLGDVLRVTGF
AtGH3.15    QEAG-----------HDPTEKPVVVDLVDVKIGHDYEPVVTTFSGLYRYRVGDVLRATGF
AtGH3.7     DK-------------------NDVVDLADVKLGCCYEAVVTNFSGLYRIRVGDILVVTGF
AtGH3.12    DK-------------------NDVVDLEDVKLGCTYEPVVTNFAGLYRMRVGDIVLVTGF
AtGH3.8     DK-------------------TEIVDLVDVKLGCYYEPLVTNHSGLHRYKMGDILLVTGF
AtGH3.18    DM-------------------NTIVDLVGVKLGCYYETVVTSYFGLHRYLIGDILQVTGF
AtGH3.19    DM-------------------ASIVDLVDVKLGCYYEPVVTNYFGLHRYLIGDILQVTGF
CpGH3.6     GVTNSINVP----KSLNEKEQQELVDLVDVKLGQEYELVVTTYAGLYRYRVGDVLRVAGF
CpGH3.5     GVTNSINVP----KSLNEKEQQELVDLVDVKLGQEYELVVTTYAGLYRYRVGDVLRVAGF
AtGH3.5     GVTNSINLP----KALTEKEQQELVDLVDVKLGQEYELVVTTYAGLCRYRVGDLLRVTGF
AtGH3.6     GVTSSISLP----KALTEKEQQELVDLVDVKLGQEYELVVTTYAGLYRYRVGDVLSVAGF
AtGH3.3     ------------------TEKSELVELADVEVGKEYELVITTYAGLNRYRVGDILQVTGF
AtGH3.2     DGAA-----------EASLDETSLVELANVEVGKEYELVITTYAGLYRYRVGDILRVTGF
AtGH3.4     DGGV-----------E----ATSLVELADVEVGKEYELVITTYAGLYRYRVGDILRVTGF
AtGH3.1     ---------------------TKAVELVDVNIGKEYELVVTTYAGLCRYRVGDILRVTGF
CpGH3.1a    PS-V-----------TR-ESSPKLVDLVDVEVGKEYELVITTYAGLYRYRVGDILRVTGF
CpCH3.1b    PSPL-----------SR-DSPPRLIDLADVEVGKMYELVITTYAGLCRYHVGDILQVTSF
AtGH3.17    HEEIHFATHSNTDDDDDALKEDLIVNLVNVEVGQYYEIVITTFTGLYRYRVGDILKVTGF
AtGH3.9     TLSF-------DLDDDEQVPCDKVVDLVNVKLGRYYELVVTTFAGLYRYRIGDVLQVAGF
CpGH3.9     MLSF-------DVVQEEEVSADKVVDLVNVKLGCYYELLVTTFAGLYHYRIGDVLQVTGF
AtGH3.10    NQS------DICIDGDFVED--KPVPLSQVKLGQEYELVLTTFTGLYRYRLGDVVEVTSF
CpGH3.10    KEDY-FNDSSPAAIDDFVEG--EPVPLSQVKVGQQYEIVLTTFTGLYRYKLGDVVEVAGF
AtGH3.11    ------------------EGEEKPVGLTQVKIGEEYEVVITNYAGLYRYRLGDVVKVIGF
CpGH3.11    HAQE-----QDLMDPDFISGEPKPVGLTEVKVGEEYEILLTNFAGLYRYRLGDVVKVMGF
                                    : *  *::*  ** ::*.. ** :  :**:: . .*
```

```
AtGH3.16     HNNAPQFYFVGRQNVVLSIDLSKTYEEDLLKAVKNAS--LLLEPHDLMLMDFTSRVDLSS
AtGH3.13     YNNSPHFRFVGRQKVVLSLHMANTYEEDLLKAVTNAK--LLLEPHDLMLMEFTSRVDSSS
AtGH3.14     YNNAPQFHFVGRQKVVLSIDMDKTYDEDLLKAVTNAS--LLLEPHDLMLMDFTSRVDSSS
AtGH3.15     YNNAPHFCFVGRQKVVLSIDMDKTYEDDLLKAVTNAK--LLLEPHDLMLMDFTSRVDSSS
AtGH3.7      HNKAPQFRFIRRDNVVLSIDLDKTNEDDLFKAVNNAK--LTLDSSHLMLIDFTSYADIST
AtGH3.12     YNNAPQFKFVRRENVVLSIDSDKTNEEDLFKAVSQAK--LVLESSGLDLKDFTSYADTST
AtGH3.8      YNNAPQFRFVRRGNLTLSIHLEITTDEDLLNAVTDAK--MVLESSNLMLMDFTSYADIST
AtGH3.18     YNNTPQFRFVRRKNIVLSVNSEATTEQDILKGLASAT--LVLESSNSMLTGFTCYADISS
AtGH3.19     YNNTPQFRFVHRKNVVLSVRSETTTEEDILKALNHVG--LVLESSDLMLMGFTCYADIST
CpGH3.6      KNKAPQFNFICRKNVVLSIDSDKTDE----------------------------------
CpGH3.5      KNKAPQFNFICRKNVVLSIDSDKTDEVELQNAVKNAV-NHLV-PFDATIADYTSYADTKT
AtGH3.5      KNKAPQFSFICRKNVVLSIDSDKTDEVELQNAVKNAV-THLV-PFDASLSEYTSYADTSS
AtGH3.6      KNNAPQFSFICRKNVVLSIDSDKTDEVELQNAVKNAV-THLV-PFDASLSEYTSYADTSS
AtGH3.3      YNSAPQFKFVRRKNVLLSIESDKTDEAELQSAVENAS-LLLG-EQGTRVIEYTSYAETKT
AtGH3.2      HNSAPQFKFIRRKNVLLSVESDKTDEAELQKAVENAS-RLFA-EQGTRVIEYTSYAETKT
AtGH3.4      HNSAPQFKFIRRENVLLSIESDKTDEADLQKAVENAS-RLLA-EQGTRVIEYTSYADTKT
AtGH3.1      HNSAPQFHFVRRKNVLLSIDSDKTDESELQKAVENAS-SILHEECGSRVAEYTSYADTST
CpGH3.1a     HNSAPQFHFVRRKNVLLSIDSDKTDEAELQRAVENAS-QLLR-EFNTSVVEYTSYADTKT
CpCH3.1b     HNSAPQFRFIRRKNVLLSIDSDKTDEAELQKGIENAS-SLLE-KYNTTVVEYTSYADTKT
AtGH3.17     HNKAPQFRFVQRRNVVLSIDTDKTSEEDLLNAVTQAKLNHLQHPSSLLLTEYTSYADTSS
AtGH3.9      YNGAPQFRFICRRNVVLSIDLDKTNEEDLHRSITLAK--KKLG-SNAFLAEYTSYADTSS
CpGH3.9      HNKAPQFRFICRRNVLLSIDNDKTNEEDLHKSITKAK--KLLDPYNALLMEYTSYADTSS
AtGH3.10     HKGTPKLSFIYRRKLILTINIDKNTEKDLQRVVDKAS-QLLSRSTRAEVVDFTSHADVIA
CpGH3.10     HNKTPKLNYICRRKLILTVNIDKNTEKDLQLVVERGC-QLLSSQTRAELVDFTSHADVVN
AtGH3.11     YNNTPQLKFICRRNLILSINIDKNTERDLQLSVESAA-KRLSE-EKIEVIDFSSYIDVST
CpGH3.11     HNSTPELKFVCRRNLVLNINIDKNTEKDLQLSVEEAG-RVLAE-EKVEVLDFTSHVDVST
              : :*.: :: * :: *.:    . :

AtGH3.16     LPGHYVLYWELGNKFKN----AKLDPKSNVLEECCLTVEESLDSIYREGRKNDKIIGPLE
AtGH3.13     FVGHYVLYWELGSKVKD----AKLEPNRDVMEECCFTVEKYLDPLYRQERRKDKNIGPLE
AtGH3.14     YPGHYVLYWELGRKVKD----AKLELDQNVLEECCFTIEESLDAVYRKGRKNDKNIGPLE
AtGH3.15     FPGHYVIYWELGSKVKD----AKFEPNRDVMEECCFTVEESLDAVYRKGRKNDKNIGPLE
AtGH3.7      IPGHYVVYWEVKNKNEDKKSKKHIELKEETFSECCLLMEDSLDSVYKICRFKEESVGPLE
AtGH3.12     FPGHYVVYLEVDTKEGEEKETAQFELDEEALSTCCLVMEESLDNVYKRCRFKDGSIGPLE
AtGH3.8      TPGHYVLYWELKAKYRN----DIVEIDKNVLVECCYVVEESLNNSYRVYRSKGGLIGALE
AtGH3.18     FPGHYVFYWELKAKDVD----DVVELDENVLEECCYALEESFDALYKRLRSKEGSIGALE
AtGH3.19     FPGHYVFYWELKAKDVQ----DVFELEEKVMVKCCSLLEESFDEVYRKNRSKDECIGPLE
CpGH3.6      ------------------------------------------------------------
CpGH3.5      IPGHYVLYWELSLNGSTPI-------PPSIFEDCCLTIEESLNSVYRQGRVSDKSIGPLE
AtGH3.5      IPGHYVLFWELCLDGNTPI-------PPSVFEDCCLAVEESFNTVYRQGRVSDKSIGPLE
AtGH3.6      IPGHYVLFWELCLNGNTPI-------PPSVFEDCCLTIEESLNSVYRQGRVSDKSIGPLE
AtGH3.3      IPGHYVIYWELLVKDQTNP------PNDEVMARCCLEMEESLNSVYRQSRVADKSIGPLE
AtGH3.2      IPGHYVIYWELLGRDQSNA-----LMSEEVMAKCCLEMEESLNSVYRQSRVADKSIGPLE
AtGH3.4      IPGHYVIYWELLSRDQSNA-----LPSDEVMAKCCLEMEESLNAVYRQSRVSDKSIGPLE
AtGH3.1      IPGHYVLYWELLVRDGARQ------PSHETLTRCCLGMEESLNSVYRQSRVADNSVGPLE
CpGH3.1a     IPGHYVIYWELLVKDPANW------PSEQVLNQCCLEMEESLNSVYRQGRVADNSIGPLE
CpCH3.1b     IPGHYVIYWELLAKDPANF------PTHEVLNQCCLAMEESLNSVYRQGRVADNSIGPLE
AtGH3.17     IPGHYVLFWELKPRHSN----DPPKLDDKTMEDCCSEVEDCLDYVYRRCRNRDKSIGPLE
AtGH3.9      VPGHYVLFWEIQG-----------HLEPKLMEECCVAVEEELDYIYRQCRTKERSIGALE
CpGH3.9      VPGHYALFWEIQFKESVVE--SVSQIDAKVIEDCCIAVEEELDYVYRRCRTNDKSVGPLE
AtGH3.10     RPGHYVIYWEIRG-----------EADDKALEECCREMDTAFVDYGYVVSRRMNSIGPLE
CpGH3.10     QPGHYIIYWEIKG-----------EVEERVLGECCCEMDASFVDHGYVVSRRSNSIGPLE
AtGH3.11     DPGHYAIFWEISG-----------ETNEDVLQDCCNCLDRAFIDAGYVSSRKCKTIGALE
CpGH3.11     EPGHYVIFWEISG-----------QVEEDVLQGCCNCLDKSFVDAGYVSSRKVNAIGPLE
```

```
AtGH3.16      IKVVKPGAFDELMNFFLSRGSSVSQYKTPRSVTHE-E-ALNILESNVVSEFLSRKTPSWE
AtGH3.13      IKVVKPGAFDELMNFFLSRGSSVSQYKTPRSVKTE-E-AVKILEANVVSEFLSQETPPWG
AtGH3.14      IKVVKPGAFDKLMNFFLSRGSSVSQYKTPRSVTNE-E-ALKILEANVVSEFLSQKTPSWE
AtGH3.15      IKVVKPGAFDELMNFFLSRGSSVSQYKTPRSVTNE-E-ALKILEANVISEFLSRKIPSWE
AtGH3.7       IKVVRQGTFDSLMDYFISQGASIGQYKTPRCIKSG-K-ALEVLEENVVATFFST------
AtGH3.12      IRVVRQGTFDSLMDFFISQGASTGQYKTPRCIKSG-K-ALQVLETCVVAKFFSI------
AtGH3.8       IRLVQQGTFDALMEFFITQGASSTQYKTPICIKST-E-ALVILEENVHACFFTDKSLSLN
AtGH3.18      IKVVQQGTFDSLMEYFISKGGSVAQYKTPMCINSS-E-TLAVLEDKVIARFYSQKSPPLN
AtGH3.19      IRVVQQGTFDSLMEYFISQGGSIAQYKTPICINSS-E-ALAVLENKVLARFFSEKSPPLD
CpGH3.6       ------------------------------------------------------------
CpGH3.5       IKIVESGTFDKLMDYAISLGASINQYKTPRCVKFA-P-IVELLNSRVTSCYFSPKCPKWV
AtGH3.5       IKIVEPGTFDKLMDYAISLGASINQYKTPRCVKFA-P-IIELLNSRVVDSYFSPKCPKWV
AtGH3.6       IKMVESGTFDKLMDYAISLGASINQYKTPRCVKFA-P-IIELLNSRVVDSYFSPKCPKWS
AtGH3.3       IRVVKNGTFEELMDYAISRGASINQYKVPRCVSFT-P-IMELLDSRVVSTHFSPALPHWS
AtGH3.2       IRVVRNGTFEELMDYAISRGASINQYKVPRCVSFT-P-IMELLDSRVVSAHFSPSLPHWS
AtGH3.4       IRVVQNGTFEELMDFSISRGSSINQYKVPRCVSLT-P-IMKLLDSRVVSAHFSPSLPHWS
AtGH3.1       IRVVRNGTFEELMDYAISRGASINQYKVPRCVNFT-P-IVELLDSRVVSAHFSPSLPHWT
CpGH3.1a      IRVVRSGTFEELMDYAISRGASINQYKVPRCVNFI-P-IMELLDSRVVSAHFSPARPHWT
CpCH3.1b      IRVVKSGTFEDLMDYAISRGASINQYKVPRCVSFT-P-IMELLDSRMVSNHFSPALPFWT
AtGH3.17      IRVVSLGTFDSLMDFCVSQGSSLNQYKTPRCVKSG-G-ALEILDSRVIGRFFSKRVPQWE
AtGH3.9       IRVVKPGTFEKLMDLIISQGGSFNQYKTPRCVKSN-SATFKLLNGHVMASFFSPRDPTWV
CpGH3.9       IRVVQPGTFEALMDLFISQRRLSQPVQNTKVHQL--------------------------
AtGH3.10      LRVVERGTFGKVAERCVGKCGGLNQFKTPRCTT--NSVMLDILNDSTIKRFRSSAYD---
CpGH3.10      LCIVETGTFKKILDYFIGNGAALSQFKTPRCTT--NKVLLTILNLCTIKRFRSTAYG---
AtGH3.11      LRVVAKGTFRKIQEHFLGLGSSAGQFKMPRCVKPSNAKVLQILCENVVSSYFSTAF----
CpGH3.11      LRLVRRGTFQKILDHYLALGAAVSQFKTPRCVGPNNNMVLQILNGNVVKSFSSTAFSSAM
```

```
AtGH3.16      LHELHSSR---
AtGH3.13      -----------
AtGH3.14      LHELHSSR---
AtGH3.15      LHELHSGR---
AtGH3.7       -----------
AtGH3.12      -----------
AtGH3.8       FSS--------
AtGH3.18      L----------
AtGH3.19      S----------
CpGH3.6       -----------
CpGH3.5       PGHKQWMNSKN
AtGH3.5       PGHKQWGSN--
AtGH3.6       PGHKQWGSN--
AtGH3.3       PERRR------
AtGH3.2       PERRR------
AtGH3.4       PERRH------
AtGH3.1       PERRRR-----
CpGH3.1a      PERRR------
CpCH3.1b      PERRR------
AtGH3.17      PLGLDS-----
AtGH3.9       P----------
CpGH3.9       -----------
AtGH3.10      -----------
CpGH3.10      -----------
AtGH3.11      -----------
CpGH3.11      CI---------
```

Fig.S3 Alignment of *C. papaya* and *Arabidopsis* GH3 proteins obtained with the ClustalW program and manual correction. Colorized shading indicated identical and conversed amino acid residues, respectively.
