# Supplementary material for: Identification, Expression and IAA-Amide Synthetase Activity Analysis of Gretchen Hagen 3 in Papaya Fruit (Carica papaya L.) during Postharvest Process
Source: Front Plant Sci. 2016 Oct 20;7:1555. doi: 10.3389/fpls.2016.01555 (PMC5071377; doi:10.3389/fpls.2016.01555)
Supplement: Supplementary file 8 [file Image4.PDF]

Fig. S4: Motif distribution analysis of CpGH3 proteins.

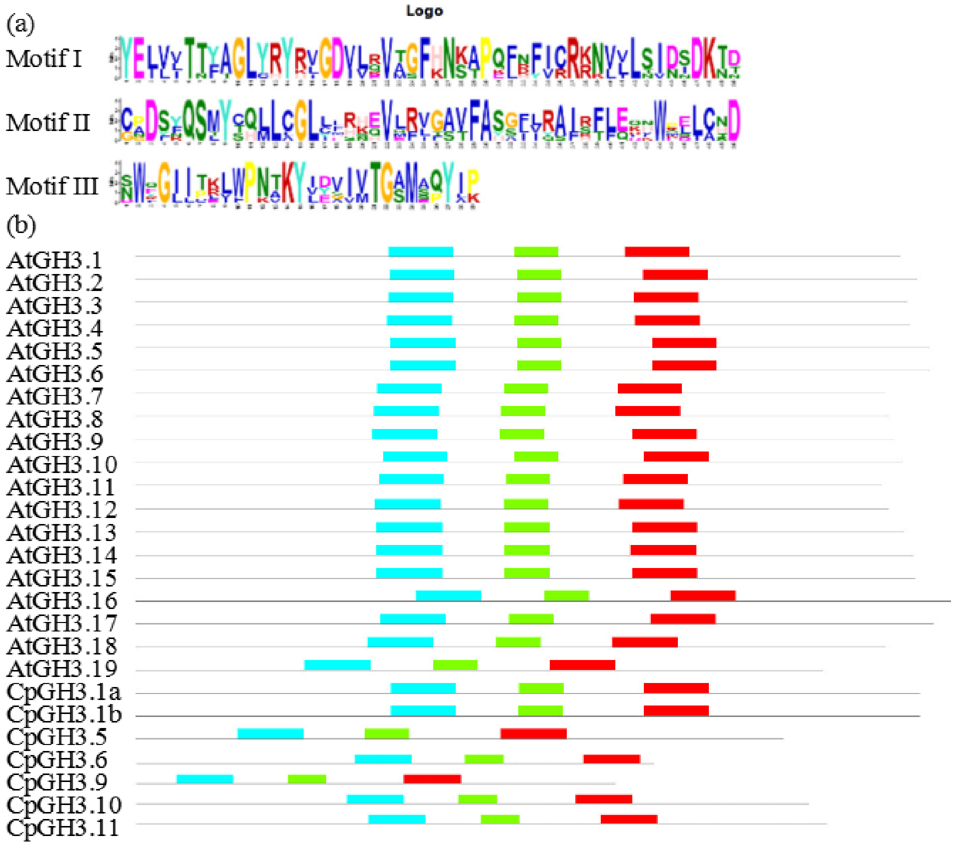

Fig. S4 **Motif distribution analysis of CpGH3 proteins.** (a) Motifs in GH3 proteins were analyzed using the MEME web server. The height of each box represents the specific amino acid conservation in each motif. (b) Three motifs representing domains I, II and III are indicated on the CpGH3 proteins by different colors.
