# Supplementary material for: Identification, Expression and IAA-Amide Synthetase Activity Analysis of Gretchen Hagen 3 in Papaya Fruit (Carica papaya L.) during Postharvest Process
Source: Front Plant Sci. 2016 Oct 20;7:1555. doi: 10.3389/fpls.2016.01555 (PMC5071377; doi:10.3389/fpls.2016.01555)
Supplement: Supplementary file 9 [file Image5.PDF]

Fig. S5 The purified GH3 proteins from *E. coli*.

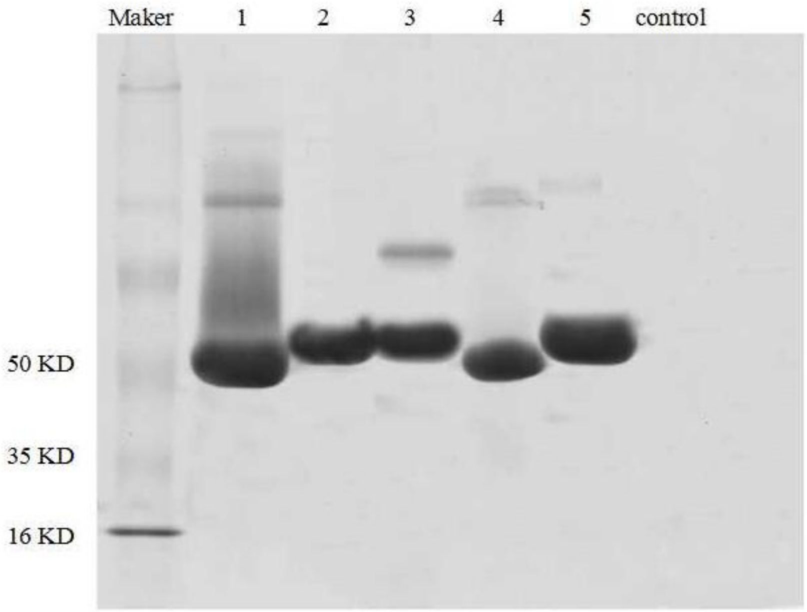

Fig. S5 The purified GH3 proteins from *E. coli*. 1: CpGH3.1a, 2: CpGH3.1b, 3: CpGH3.5, 4: CpGH3.6, 5: CpGH3.9. Control: negative control.
