# Supplementary material for: Identification, Expression and IAA-Amide Synthetase Activity Analysis of Gretchen Hagen 3 in Papaya Fruit (Carica papaya L.) during Postharvest Process
Source: Front Plant Sci. 2016 Oct 20;7:1555. doi: 10.3389/fpls.2016.01555 (PMC5071377; doi:10.3389/fpls.2016.01555)
Supplement: Supplementary file 10 [file Image6.PDF]

Fig. S6 The production rate of ethylene measurements.

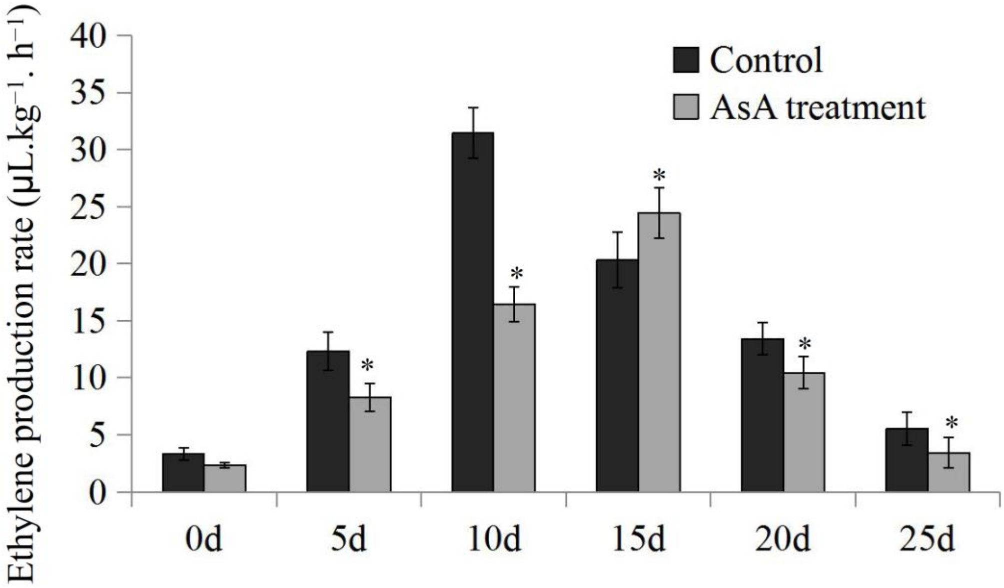

Fig. S6 **The production rate of ethylene measurements.** The differences in production rate of ethylene between the controls and AsA-treated fruits during the postharvest process. The data were analyzed by five independent repeats, and standard deviations were shown with error bars. The significant ($P < 0.05$) differences between the controls and AsA treatments are indicated by an asterisk.
